# Sericin Electrodes with Self-Adhesive Properties for Biosignaling

**DOI:** 10.1021/acsbiomaterials.4c02234

**Published:** 2025-02-04

**Authors:** Davide Vurro, Aris Liboà, Ilenia D’Onofrio, Giuseppe De Giorgio, Silvio Scaravonati, Marco Crepaldi, Alessandro Barcellona, Corrado Sciancalepore, Vardan Galstyan, Daniel Milanese, Mauro Riccò, Pasquale D’Angelo, Giuseppe Tarabella

**Affiliations:** † Institute of Materials for Electronics and Magnetism (IMEM-CNR), Parco Area delle Scienze 37/A, Parma 43124, Italy; ‡ Department of Chemistry Life Sciences and Environmental Sustainability, University of Parma, Parco Area delle Scienze 17/A, Parma 43124, Italy; § Department of Mathematical, Physical and Computer Sciences, University of Parma, GISEL & INSTM, Parco Area delle Scienze 7/A, Parma 43124, Italy; ∥ Electronic Design Laboratory, Fondazione Istituto Italiano di Tecnologia, Via Enrico Melen 83, Genova 16152, Italy; ⊥ Department of Engineering for Industrial Systems and Technologies, University of Parma, Parco Area delle Scienze 181/A, Parma 43124, Italy

**Keywords:** biocompatible materials, silk sericin, epidermal electrodes, electrocardiography, wearable electronics

## Abstract

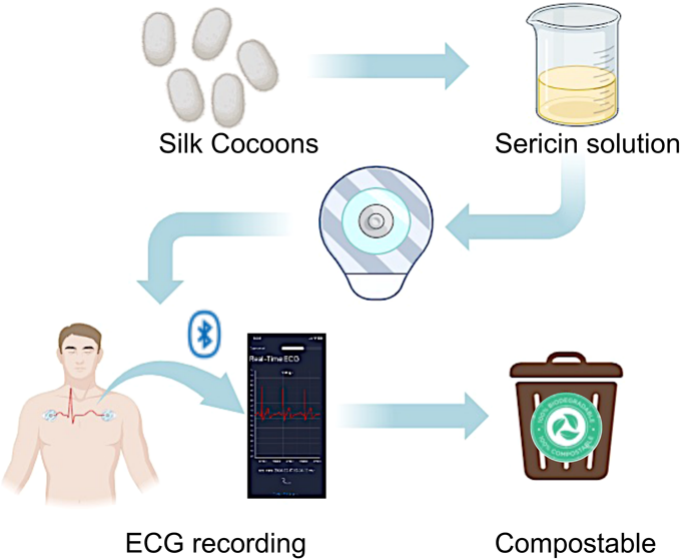

The combination of green manufacturing approaches and bioinspired materials is growingly emerging in different scenarios, in particular in medicine, where widespread medical devices (MDs) as commercial electrodes for electrophysiology strongly increase the accumulation of solid waste after use. Electrocardiogram (ECG) electrodes exploit electrolytic gels to allow the high-quality recording of heart signals. Beyond their nonrecyclability/nonrecoverability, gel drying also affects the signal quality upon prolonged monitoring of biopotentials. Moreover, gel composition often causes skin reactions. This study aims to address the above limitation by presenting a composite based on the combination of silk sericin (SS) as a structural material, poly(vinyl alcohol) (PVA) as a robustness enhancer, and CaCl_2_ as a plasticizer. SS/PVA/CaCl_2_ formulations, optimized in terms of weight content (wt %) of single constituents, result in a biocompatible, biodegradable “green” material (free from potentially irritating cross-linking agents) that is, above all, self-adhesive on skin. The best formulation, i.e., SS(4 wt %)/PVA(4 wt %)/CaCl_2_(20 wt %), in terms of long-lasting skin adhesion (favored by calcium-ion coordination in the presence of environmental/skin humidity) and time-stability of electrode impedance, is used to assemble ECG electrodes showing quality trace recording over longer time scales (up to 6 h) than commercial electrodes. ECG recording is performed using customized electronics coupled to an app for data visualization.

## 1 Introduction

Waste management is one of the major challenges of our time. Challenges like renewable resources, circular economy, and biodegradation are becoming increasingly prominent. In this context, materials science has set the objective of developing new solutions aimed at meeting the requirements of a circular economy and sustainability. Many researchers, in fact, are dedicating their efforts to discovering new biodegradable materials and innovative techniques for waste reduction. A substantial amount of waste generated in daily life consists of MDs. In Europe alone, half a million MDs are available, and 90% of these are disposable, contributing to the overall increase in solid waste.^1^ In particular, electrodes for electrophysiology recording, such as electrocardiogram (ECG) electrodes, represent a large slice of disposable MDs massively used in medical clinics.

Among commercial types of electrodes for ECG, gelled Ag/AgCl electrodes (i.e., wet-type electrodes) are the most common ones due to their ease of use and affordability.^2^ The widespread use and disposal of these electrodes pose a significant environmental challenge due to the difficulty of managing nondegradable residues. Another concern regards the signal stability over time that they may guarantee. The reliance of gelled electrodes on electrolyte gels can indeed lead to degradation of signal quality over time. Specifically, while the gel component of ECG electrodes enhances adhesion and improves signal quality upon short monitoring, as the gel dries, the electrode impedance increases, potentially affecting the accuracy of ECG recordings during long-term monitoring. Gels can also contribute to skin irritation during prolonged exposure. This is particularly true for individuals with sensitive skin, and it has been also demonstrated in patients undergoing long-term ECG monitoring.^3,4^ In response to the limitations of wet electrodes, researchers have focused their efforts on developing dry electrodes. These electrodes aim to provide high-quality ECG signals over extended monitoring periods, minimizing motion artifacts and reducing skin irritation.^2^ Polymer composite skin-contact electrodes are emerging as a promising alternative to traditional gel-based ones. These materials offer several advantages. First, their synthesis is straightforward and cost-effective, and they possess high mechanical stability, allowing them to conform to body movements and reduce motion artifacts. Additionally, polymer composites are biocompatible and biodegradable, ensuring safety for the human body and easy disposal after use with minimal environmental impact, respectively.

In the context of eco-friendly fabrication, waste management, and circular economy, recently SS is experiencing extensive use in several innovative biotechnological applications. Silk sericin (SS) is a major byproduct of silk production (accounting for approximately one-eighth of the dry cocoon’s weight), and when discarded in effluents, it may induce environmental issues.^5^ However, to reduce the accumulation of sericin in the environment and harness its potential, it is recycled and utilized in various biotechnological applications, such as carbonaceous ink stabilizer for electronic and electrochemical sensors^6−8^ as a structural material for drug delivery,^9,^ tissue engineering,^11−13^ and food packaging.^14−16^

SS is a water-soluble protein that acts as a natural adhesive, binding together the two silk fibroin (SF) strands within silk fibers.^17^ This protein comprises 20–30% of the total silk composition.^18^ SS consists of 18 amino acids, featuring a high concentration of polar side groups, such as hydroxyl and carboxyl groups. There are five distinct types of SS, designated as SS1 to SS5,^19^ with molecular weights ranging from 20 to 400 kDa.^20^ SS represents a perspectival biomaterial with antioxidant, anti-inflammation, biodegradability, cell growth, and ultraviolet (UV) protection properties.^21^ All of these aspects can be tuned and emphasized by changing the amino acid sequences of SS, which may be done upon choosing the proper extraction method and, thanks to the presence of hydroxyl and carboxyl groups, by combining it with other materials such as metal nanoparticles or synthetic polymers.^22−25^

While SS inherently possesses biocompatibility and biodegradability, its pure form exhibits low mechanical properties. Indeed, different studies on several silkworm varieties have shown that tensile strength can range from 10 to 50 MPa, while elongation at break can vary from a minimum of 1.4 to 3.6%. The Young’s modulus, indicative of the material’s stiffness, can range from around 1000 to 1800 MPa. These parameters can be enhanced through crystallization treatments, such as the use of formic acid, which promotes β-sheet structure formation.^26,27^ For this reason, SS requires the addition of polymers with specific properties, plus cross-linkers, to achieve stable material structures such as films, membranes, and hydrogels.^28,29^ Several biocompatible polymers like poly(vinyl alcohol) (PVA),^30−32^ bacterial cellulose,^33,34^ chitosan,^35,36^ collagen,^37^ and SF^38^ were reported as SS mechanical robustness enhancers. Among these, PVA has been extensively utilized in the development of films, scaffolds, and membranes for biomedical and bioelectronics applications due to its biocompatibility, biodegradability, and film-forming properties.

Herein, we propose a methodology aimed at promoting the development of an SS-based film that is biocompatible, biodegradable, manageable, unobtrusive, and self-adhesive to the skin and, hence, suitable for epidermal electrode applications. The film incorporates PVA and CaCl_2_ to enhance its mechanical properties and hygroscopic behavior, respectively, resulting in a “green” self-adhesive material with stable and long-lasting skin adhesion. PVA is often used with SS in formulations with cross-linking agents^13,39,40^ or fabrication techniques.^31,41,42^ Our approach focuses on additive-free SS/PVA blends, but this can lead to phase separation.^23^

To overcome this issue, the hygroscopic salt CaCl_2_ was added to the SS/PVA blend as a plasticizer.^43,44^ Film properties have been characterized through attenuated total reflection Fourier transform infrared (ATR-FTIR) spectroscopy, Scanning Electron Microscopy (SEM), electrochemical impedance spectroscopy (EIS), and stress–strain analysis to investigate chemical, morphological, electrical, and mechanical properties, respectively. Skin attachable electrodes based on the combination of Ag/AgCl snap buttons and SS/PVA/CaCl_2_ films have been fabricated and characterized in terms of their interaction with skin upon acquiring impedance values. Finally, different electrode combinations in terms of blend percentage composition were employed for ECG monitoring, using customized wearable reading hardware equipped with a dedicated iOS app, to disclose the blend formulation capable of guaranteeing a prolonged efficiency under long-lasting and continuous data acquisition. Our results offer a cue toward solutions responding to the need for long-lasting monitoring in various contexts, for instance, in medical settings in case of patients showing scarce cooperativity and poor tolerance to long-lasting monitoring sessions, as it may happen in case of pediatric patients,^45^ but also in sports or working settings where workers face strenuous activities.^46^

## 2 Materials and Methods

### 2.1 SS Extraction

SS powders have been prepared using the autoclave degumming method.^47^ 50g of silk cocoons have been weighed and cut into small pieces. The cocoons were then immersed in 1L of ultrapure water and placed in an autoclave. The degumming process has been conducted at 120 °C for 1 h. The degumming process dissolves only the SS layer, leaving behind the solid SF. The SS solution has been lately collected, lyophilized (FreeZone 1 Liter, Labconco corporation, Kansas City, MO) to obtain dry powders, and stored at −18 °C. Before use, the SS is dissolved in water and centrifuged (8000 rpm, 10′) to remove any impurities that may be present. Subsequently, the concentration is determined and adjusted for the next processing stages.

### 2.2 Fabrication of Plasticized SS Films

Plasticized SS-based films have been prepared by mixing the SS aqueous solution and a PVA (115000, purity 88%, VWR) aqueous solution in the presence of a variable concentration of CaCl_2_ (Sigma-Aldrich). SS powders have been dissolved in Milli-Q water at 60 °C obtaining a final concentration of 8 wt %. A different amount of salts (10, 20, 30 wt % with respect to SS weight) has been added to the solution and stirred until complete dissolution of SS. 10 wt % PVA solution has been subsequently added to the SS/CaCl_2_ solution under stirring in a ratio 1:1 v/v. The obtained solution has a final concentration of 4 wt % of SS and 4 wt % of PVA. The solution has been thermalized and drop-cast in a custom TEFLON mold (1.5 mL for each pod), leaving it to dry overnight under a hood. The mold, in particular, is composed of 64 circular pods (2.5 cm in diameter) with a removable bottom layer. The bottom layer height has been fixed to 0.3 mm to avoid loss of materials over mold edges during the drying process. Dried films have been peeled off and stored in a Petri dish until use.

### 2.3 Chemical and Morphological Analysis

Chemical analysis of the SS/PVA/CaCl_2_ films with different salt concentrations was conducted using ATR-FTIR spectroscopy. SS and PVA powders have been directly analyzed, while SS/PVA/CaCl_2_ films were cut into small pieces and dried before measurements. The spectra have been collected using an Agilent Cary 630-FTIR spectrometer (Agilent Technologies, Santa Clara, CA) equipped with an ATR module in the wavenumber range of 500 to 4000 cm^–1^. The instrument resolution is ≤2 cm^–1^. The morphology and surface of the plasticized films were examined by acquiring morphological images using a Zeiss Auriga compact field emission scanning electron microscope (Karl Zeiss). The magnification (Mg), electron high tension (EHT), and working distance (WD) used for SEM image acquisition were 2.00 KX, 5.00 keV, and 7.5 mm, respectively. Samples were cut into small pieces and coated with a thin sputtered gold layer (∼10 nm) prior to SEM analysis.

### 2.4 Ionic Conductivity

EIS measurements have been performed in the frequency range 5 Hz–13 MHz using an HP 4192A impedance analyzer, by applying a 0.1 V amplitude AC signal. Humidity-controlled measurements were performed with a customized humidity-controlled chamber. RH has been controlled with a humidity sensor and two independent inlets: one injecting pure N_2_, the other insufflating water saturated N_2_.

The system consists of an ionic conductor disk between two blocking (impermeable to the ions of the ionic conductor) metal foam electrodes, acting as current collectors and allowing the exchange of humidity between the surface of the disc and the atmosphere in the chamber.

This system can be represented with the equivalent circuit model illustrated in the inset of Figure 4A, in which the resistance *R*_i_ represents ions crossing the electrolyte, and *R*_e_ represents the electronic resistance of the experimental system, such as contacts and cables. It was demonstrated, both experimentally and theoretically, that the interface between a solid electrolyte and a purely electronic conductor can be modeled as a simple series of a capacitor (*C*_int_) and an ionic resistance (*R*_i_), representing the accumulation of ions at the metal/electrolyte interface. In a nonideal system, a constant phase element (CPE) is used in place of a capacitor *C*_int_. Finally, considering that the system consists of two metal electrodes separated by a dielectric, a geometric capacitor, *C*_geom_, is added in parallel to the model.

### 2.5 Mechanical Measurements

Tensile characterizations have been conducted on plasticized films with three different salt concentrations to determine the Young’s modulus and elongation at break values. SS solutions with 10, 20, and 30 wt % have been drop-cast onto 10 cm polystyrene Petri dishes and allowed to dry overnight. After drying, the films were peeled off and die-cut into dumbbell specimens, type 5A, according to the standard ISO 527. The samples have been humidified for 1 h and then left at a controlled relative humidity for an additional 15 min before measurements. Stress–strain curves have been obtained by using a TesT dynamometer (TesTWinner Gmbh, model 112) equipped with a load cell of 2 kN and pneumatic clamps to prevent the specimens from slipping out during measurements. The tests were conducted at a deformation speed of 50 mm/min and a pretension of 0.5 N. The experiment continued until the sample broke. The experiments were repeated for two samples for each salt concentration in the blend. All of the experiments were conducted with an environmental humidity equal to 60%.

### 2.6 Electrode Fabrication and Impedance Skin/Electrode Measurement

The proposed electrode consisted of three layers: a commercial Ag/AgCl snap button, a cellulose layer, and an SS/PVA/CaCl_2_ film. Before electrode fabrication, the cellulose layer has been cut into a circular shape with a diameter of 2.5 cm. A hole of 0.4 cm has been made in the center to accommodate the commercial Ag/AgCl snap buttons. The SS/PVA/CaCl_2_ films were humidified for 1 h in a custom-designed climatic chamber before the final assembly of the devices. This step allowed the films to absorb moisture and become adhesive. After humidification, the cellulose/Ag/AgCl layer was glued to the SS/PVA/CaCl_2_ film using the adhesive properties of the SS layer. The assembled devices were then stored in dry conditions until their use.

The amplitude, or modulus of complex impedance, has been measured as a function of frequency using an electrochemical interface (PalmSens 4) with the electrodes positioned in a typical three-electrode configuration on the right arm of a volunteer.^48^ The selected volunteer, a Caucasian male, 41 years old with no history of heart disease, gave written, informed consent before participation to on-body measurements. In this configuration, our device served as the working electrode WE, while commercial gelled Ag/AgCl electrodes were used as the counter electrode (CE) and reference electrode (RE) electrodes for all measurements. Before EIS measurements, the SS/PVA/CaCl_2_ films were humidified for 1 h in a custom climatic chamber and placed on the right forearm, 30 cm away from the CE positioned above, on the arm, and 5 cm away from the RE (right wrist). These distances were maintained constant throughout all of the experiments. All working electrodes (Ag/AgCl, SS/PVA/CaCl_2_ 10 wt %, SS/PVA/CaCl_2_ 20 wt %, and SS/PVA/CaCl_2_ 30 wt %) have been simultaneously tested to ensure consistent relative humidity conditions during the experiments. The amplitude of the complex impedance has been recorded over a frequency range from 0.1 Hz to 100 kHz at different times (*t* = 0, *t* = 3 h, and *t* = 6 h) in order to evaluate the time dependence of the impedance modulus. Five plots were recorded for each electrode under analysis and at each selected time.

### 2.7 ECG Reader Hardware

The system comprises a commercial AD8236 micropower instrumentation amplifier with zero crossover distortion (Figure 6A), and the signal amplification front-end has been modified to meet a single power supply and operation with two electrodes.^49^ To implement a two-electrode signal readout with a single-ended supply, we have implemented a supply voltage divider (active, OpAmp-based) that generates a reference voltage *V*_REF_ of 1.65 V, based on the single supply of *V*_DD_ = 3.3 V.

Moreover, the reference voltage is brought at a higher impedance than the skin-electrode interfaces with two equal resistors *R*_E_. The resistors *R*_B_ in the reference voltage generators have a nominal value of 100 kW, and the filtering capacitor *C*_B0_ has a nominal value of 10 nF, with the resistor implementing a low-pass filter with a 160 Hz cutoff frequency. The components *R*_B0_, *R*_B1_, *C*_B1_, and *C*_B2_ (nominal values 1kΩ, 100 Ω, 100pF, and 1 μF) are used to limit the impact of the OpAmp noise to the output. The voltage *V*_REF_ is used as a reference for the amplification stages. The AD8236 gain resistor *R*_G_ value is 470 kW, which leads to a differential gain of 5.9. The reference terminal of the instrumentation amplifier is fed by an integrator, implemented using an AD8603, the OpAmp used in all active circuits of this system. Such an integrator is useful for implementing an AC (high pass) filter and rejecting differential-mode offsets. In this prototype, the integration capacitor CR is 1mF, while the feedback resistor *R*_R_ is 100kΩ. The value of *C*_R_ has been decreased from the original 4.7 μF value from the reference design to avoid stability issues while fixing the common mode of the electrodes through *R*_E_. After differential amplification, the ECG signal is processed by the second stage, a noninverting amplifier, with a nominal gain of 221. The nominal values of *R*_0_ and *R*_1_ are 1 and 220kΩ, respectively. The overall gain of the front-end is then 1300, which is enough to bring a 1 mV-order ECG signal from the electrodes to 1.3 *V*_pp_ for further filtering and last wireless transmission. The last stage of the front -end comprises a second-order Bessel filter with a cutoff frequency of 46 Hz (slightly modified according to the reference design to standard E12 series components), as presented in the reference design. The nominal values of the components *R*_2_, *R*_3_, *C*_0_, and *C*_1_ are 22kΩ, 3.6kΩ (three 1kΩ in series with two parallel 1.2kΩ resistors), 220nF, and 680nF. Before ADC conversion, a low-pass filter with a cutoff frequency of 482 Hz is implemented using *R*_4_ and *C*_2_, of nominal value 330 Ω and 1 μF, respectively. The capacitor *C*_2_ operates also as a charge reservoir for a possible downstream switch capacitor analog-to-digital converter (ADC).^49^

The resulting amplified and filtered ECG signal *V*_OUT_ is fed to the pin **PA4** pin of a Seeeduino Xiao nRF52840 board, which comprises a Bluetooth 5.0 transceiver and an ARM Cortex M4 CPU with a floating point unit (FPU).^50^ The nRF52840 module acquires also the reference voltage *V*_REF_ generated by the analog electronics for further expandability and to implement basic checks on the operation of the front end to identify malfunctions. In this prototype, however, this input was not used.

The module comprises a diode-based interface to a 5 V inductive power module and a 500 mA Adafruit wireless charging kit.^51^ As in this project, the Seeeduino Xiao module is considered a part, and thus, no internal modifications of the module are performed, the voltage obtained with the wireless charging module is filtered using a ferrite F_1_ (220W, at 100 MHz) to avoid noise injection on the Xiao module, and it activates a HSMH-C190 red LED D_0_ biased through *R*_L_ (100 Ω nominal value). The power signal is used as a Universal Serial Bus (USB) power source (**VBUS** on the Seeeduino Xiao), *V*_CC_ in the schematic. Power conflicts between the wireless charging module and the USB power are prevented using Schottky diode D_1_, which ensures current delivery to **VBUS** only if the USB port of the Xiao module is not powered. To provide a clean voltage supply to the analog module, thus not using the internal Seeeduino regulator, which is used to power the nRF5280, we have used an external LP38692MP-3.3 linear regulator, which converts the raw battery voltage *V*_BATT+_ into 3.3 V *V*_DD_.

### 2.8 Firmware

The ECG reader firmware has been developed by employing the Arduino integrated design environment (IDE) using the provided default libraries. The Bluetooth connection interval is set at 18.75 ms, and the bit rate is set at 2Mb/s. The 12-bit internal successive approximation ADC (SAADC) of the Seeeduino Xiao module is used through a direct memory access (DMA) so as not to impact the CPU time, with an oversampling rate of 256 and oversampling frequency of 51.2 kHz, resulting in an ECG sample rate of 200 Hz. Conversion is initiated by setting a specific bit of the ADC register. Observing the maximum MTU size negotiated with an iPhone XR used for the experiments, we assumed a maximum payload size of 120 bytes.

The operation of the main loop is minimal and straightforward. The system waits for a new Bluetooth connection. If a Bluetooth connection is absent, it waits until the connection is established. Once connected, the main loop periodically invokes a series of ADC conversions and streams data with Bluetooth. In particular, the system invokes a cycle of ADC reads using DMA lasting 20 subsequent reads. The main loop waits for a flag to be set that indicates that the conversion is finished. If finished, the 20 integer values of the ADC are stored in an array with separator ″**:**″ (except for the last symbol), for instance, “1230:1000:0998:1232.” With such data formatting, the obtained string reaches 99 bytes (20 × 8 + 19), consistently below the maximum transmission unit (MTU) size of 120. The remaining bytes in the MTU can be devoted to transmitting other information, such as battery level and other diagnostic information. However, this implementation did not transmit data other than the ECG values. The integer data transmitted by the device is converted by the iOS application into voltage units once received by knowing the ADC input range (3.3 V) and the resolution N (12 bit), i.e., by multiplying the integer data by the constant 3.3/(2^N^ – 1). Data are streamed in chunks of 20 values until the Bluetooth transceiver is disconnected. Observe that all of the CPU operations, including conversion, flag set, and Bluetooth transmission, are completed in less than 1:200 Hz, i.e., 5 ms, to avoid losing samples between distinct ADC chunks. The code in the main loop must have a well-determined runtime under any condition. When the Bluetooth transceiver is disconnected, the conversion is stopped, and the system waits for a new connection. To enable maximum simplicity, the system does not include any power supply button. When the battery is over, the CPU stops, and the system turns off until power is restored using the wireless charging port.

### 2.9 Physical Design and Mechanical Enclosure

The schematic in the inset of Figure 6B has been designed using KiCad 8.0, and a custom printed circuit board (PCB) has been implemented. The PCB, shown in Figure 6B, has been fabricated using a rapid prototyping LPKF Protomat S104, using two-layer FR4, and the components are soldered in-house. The contour of the PCB has been adapted to be hosted in a commercial enclosure Minitec B9004907, size M. However, the lateral mechanical closure part has been widened to host both the battery and the wireless charging receiver (the white part in Figure 6B). Such a part has also been modified to provide holes to hold the two electrode wires shown in the figure.

### 2.10 iOS Application

To read the continuous ECG signals from the reader and save raw data, thus enabling extensive measurement sessions, we implemented an iOS 17 application using Xcode in the Swift programming language. A screenshot of the main window is shown in Figure 6D. The application enables a user to connect to the device by means of the iPhone Bluetooth chip and to continuously save CSV data of the raw ECG signals on the device. These data are then easily transferred to other computers to enable their analysis. The application works in background mode and uses the standard Apple libraries and an additional Core Plot library for real-time plotting of the signals.^52^ The application saves raw data in CSV files (with time and date corresponding to the first sample in its file name) lasting 1 min each and not in a single CSV file to avoid potential data losses in case of software crash or abrupt device disconnections, depending on the current wireless network load. As the ECG reader prototype does not implement timestamping, the app also indicates the current packet rate as a gauge bar to inform users of missed packets. To enable default connections to a given device, the configuration screen, not shown here for brevity, permits the user to select the default ECG reader device to connect. The setting is written locally in a file in the application folder and is recalled, if present, and read every time the application starts. The application is finally deployed in an iPhone XR and used for the experiments.

### 2.11 ECG Measurements

ECG acquisitions have been carried out using custom ECG reader hardware designed to collect the signal with a two-electrode setup (common ground). Electrodes were positioned near the shoulders of the selected volunteer at a distance of 30 cm (Figure 6C). For each pair of electrodes (commercial Ag/AgCl, SS/PVA/CaCl_2_ 10 wt %, SS/PVA/CaCl_2_ 20 wt %, SS/PVA/CaCl_2_ 30 wt %), the distance was maintained constant, and the acquisition time was set to 1 h. The ECG reader saves data every 20 s of acquisition and streams them to a mobile device equipped with an iOS app developed for visualization of ECG traces. QRS peak amplitude analysis has been conducted using the licensed data analysis software OriginLab Pro 2019. The best-performing SS-based electrode has been selected for long-term monitoring (up to 6 h) using the same configuration, on the same subject.

## 3 Results and Discussion

### 3.1 SS Film Fabrication and Chemical and Morphological Characterization

Table 1 reports the best composition of the proposed standalone blend. The reported compositions have been selected among several of the three components (reported in Table S1) and tested to find the optimal composition targeted to the achievement of the desired adhesion properties.

**Table 1:** SS/PVA/CaCl_2_ Blend has the Best Composition: Concentration Ratios, wt %, are Related to the Weight Content of the Reported Components of the Blend Composite

SS (wt %)	PVA (wt %)	CaCl_2_ (wt %)
4	4	10
4	4	20
4	4	30

The mixing between SS and PVA solutions at a concentration ratio of 4% (corresponding to 1:1 v/v mixing) overall yielded the best manageability of related films. An excess of PVA with respect to SS resulted in a rigid film with scarce intrinsic adhesion, while an excess of SS has been found to determine an extremely soft and swellable film. Various percentages of CaCl_2_ based on the SS weight content were tested, with 10, 20, and 30% showing the most promising self-adhesion of SS/PVA/CaCl_2_ films to the skin. The chemical structure of SS/PVA/CaCl_2_ films has been characterized by ATR-FTIR spectroscopy.

**Figure 1:**
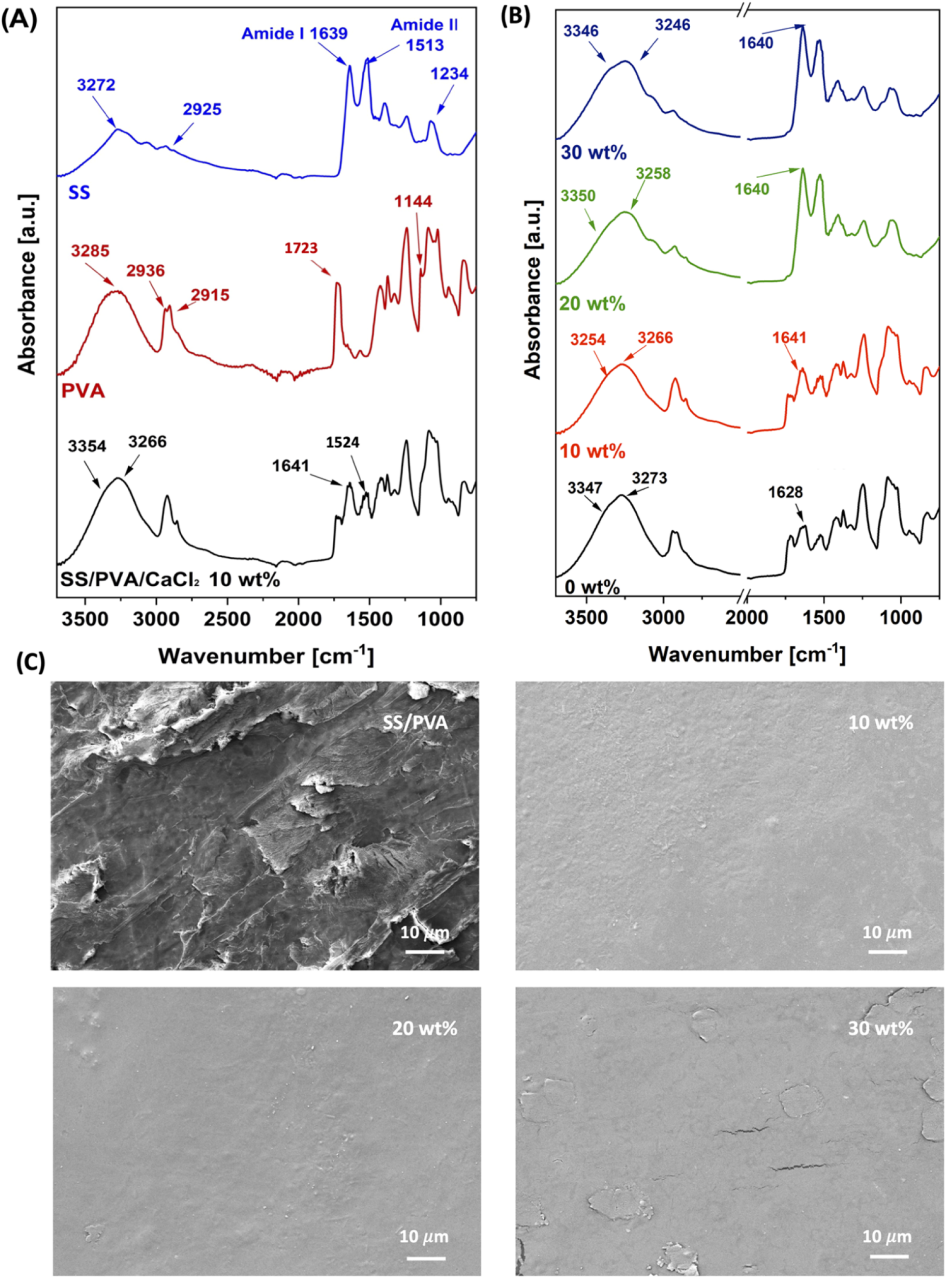
(A) Comparison between the FTIR spectrum of pure SS (blue curve), pure PVA (red curve), and SS/PVA/CaCl_2_-based film (black curve) with a salt weight percentage content of 10%; (B) comparison among FTIR spectra of SS/PVA/CaCl_2_ blends at different contents of CaCl_2_ (i.e., 0, 10, 20, and 30 wt %); (C) SEM images of SS/PVA and SS/PVA/CaCl_2_ at different CaCl_2_ concentrations, as reported above.

Figure 1A reports a comparison of the FTIR spectra for pure SS, pure PVA, and SS/PVA/CaCl_2_ blend with 10 wt % of CaCl_2_. The spectrum acquired for pure SS (blue curve) shows peaks at 1639, 1513, and 1234 cm^–1^, corresponding to the amide I (1700–1600 cm^–1^), amide II (1600–1500 cm^–1^), and amide III (1400–1200 cm^–1^) groups, respectively.^24,53^ These groups are related to C=O stretching (amide I), N–H bending, and C–O stretching (amide II), and both C–N stretching and C=O bending vibration (amide III).^54,55^

The amide I band is particularly useful for determining the crystallinity of protein structures. It is sensitive to the hydrogen bond pattern between C=O and N–H residues, dipole–dipole interactions, and the geometry of the polypeptide backbone.^56^ Formation of hydrogen bonds influences the peak position of C=O vibration, providing information about the main conformation of the protein.^57^ The peak located at 1639 cm^–1^ in pure SS spectra reflects the random coil-dominated structure of the protein.^58^ While the SS/PVA/CaCl_2_ spectrum (black curve) also exhibited these amide groups, a blue shift has been observed in amide I and amide II (to 1641 and 1536 cm^–1^, respectively) due to the presence of calcium ions. The calcium ions intercalate into the protein structure and interact with carboxyl (−COOH) and amino (−NH_2_) groups through chelation.^59^ The amide III group was not discernible in the blend film due to overlap with the PVA signal. In the region between 3600 and 2700 cm^–1^, a stronger absorption band associated to O–H and −N–H stretching has been observed. An intense peak at 3272 cm^–1^ is indicative of hydrogen bonding in β-sheet structures, while a secondary peak at 2925 cm^–1^ is related to the −CH groups of the SS protein.^60^ In SS/PVA/CaCl_2_ blends, the peak at 3272 cm^–1^, related to SS, is overlapped with the broader peak of PVA at 3285 cm^–1^ (O–H stretching). This peak red-shifted due to the interaction between Ca^2+^ ions and amine groups and the formation of hydrogen bonds during the reaction.^44,59^ A shoulder at 3354 cm^–1^ (stretching of O–H groups of water molecules) also appeared, demonstrating the ability of Ca^2+^ ions to capture environmental humidity.^59^ The FTIR spectrum of pure PVA (red curve) has shown the characteristic peaks of this polymer, which are at 3285, 2936, 2915, 1428, 1370, 1144, and 1086 cm^–1^ and are related to O–H stretching, asymmetric and symmetric CH_2_ stretching, CH_2_ bending, −CH wagging, C–O stretching, and O–H bending, respectively.^60^ The peak centered at 1723 cm^–1^ (C=O stretching) is related to the presence of poly(vinyl acetate) (PVAc) in the pure PVA due to the 88% purity of the chemical used in this study. Most of all of these peaks are present in the FTIR spectrum of blend-based films, demonstrating the successful blending between SS and PVA. The formation of hydrogen bonds between the two polymers in the blend films is demonstrated by the disappearance of the peak at 1144 cm^–1^ that indicated a reduction in the crystallinity fraction of PVA.^60^ Also, the acetate group peak decreases in intensity in the SS/PVA/CaCl_2_ blends due to the chelation between the carboxylate group of PVA and Ca^2+^ ions. Figure 1B shows the comparison of the FTIR spectrum of SS/PVA/CaCl_2_ blends at different concentrations of the calcium chloride salt. The peak at 3266 cm^–1^ red-shifted upon increasing the salt concentration, due to the formation of strong interaction between CaCl_2_ and PVA molecules.^44^ Furthermore, the peak at 3254 cm^–1^ becomes even more evident as the Ca^2+^ concentration increases, demonstrating the attitude of the blend at efficiently coordinating water molecules.^59^ In the SS/PVA blend, the amide I band exhibits a red shift and a decrease in intensity due to the formation of hydrogen bonds between the two polymers.^60^ Upon salt addition, the amide I band undergoes a blue shift from 1628 to 1641 cm^–1^, attributed to the disruption of hydrogen bonds and the chelation of Ca^2+^ ions with carbonyl residues. Interestingly, after the addition of 20 and 30 wt % of salt, an increase in intensity is observed, suggesting an increase in the random coil conformation of sericin.^59,61^

SEM analysis has been carried out to evaluate the morphological characteristics of the proposed films. Figure 1C shows SEM micrographs of the synthesized SS/PVA and SS/PVA/CaCl_2_ films with varying salt concentrations (i.e., 10, 20, and 30 wt %). The SEM images show smooth and uniform surfaces without phase separation, indicating that the proposed method enables the formation of a homogeneous blend between SS and PVA. Furthermore, the addition of CaCl_2_ effectively prevents phase separation between SS and PVA during the drying process. Cracks over the surface of the film with 30 wt % of CaCl_2_ are related to the drying process during gold sputtering before the SEM analysis.

### 3.2 Mechanical Measurements

**Figure 2:**
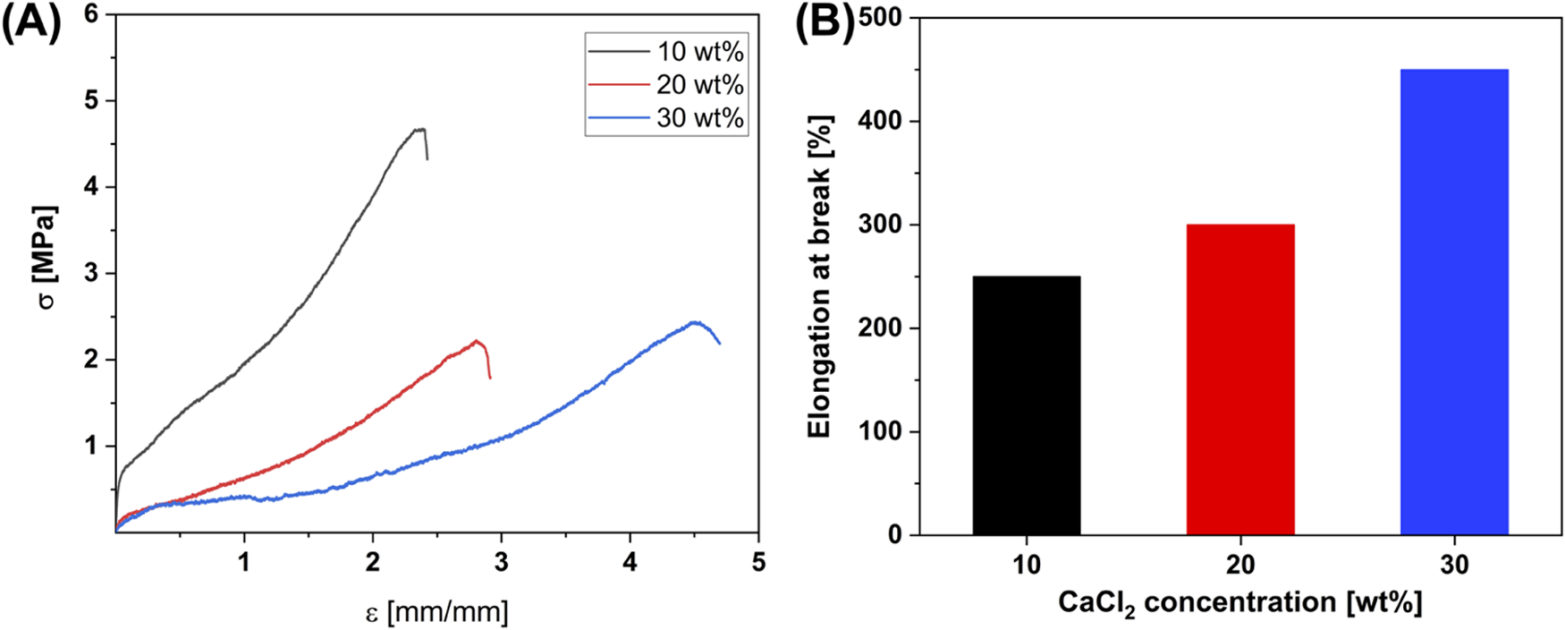
(A) Stress–strain curves of SS/PVA/CaCl_2_ blend-based films at different CaCl_2_ concentrations (i.e., 10, 20, and 30 wt %). (B) Dependence of elongation at break on CaCl_2_ weight percentage content in SS/PVA films.

The tensile mechanical characterization of as-produced films with varying concentrations of CaCl_2_ has been performed by recording stress (σ)-strain (ε) curves, reported in Figure 2A,B.

The CaCl_2_ concentration exerts a significant influence on the mechanical behavior of the specimens. Indeed, in SS/PVA/CaCl_2_, at 10%, the typical features of tough materials, with a large strain (250%) and higher elastic modulus (about 57 MPa), are observed. In SS/PVA/CaCl_2_, at 20%, the strain at break increases (300%) while the stiffness is reduced to 11 MPa. SS/PVA/CaCl_2_ 30% shows an increasingly elastomer-like behavior, with the highest elongation at break of 450% and the lowest elastic modulus of about 3 MPa. Thus, as also demonstrated in the case of SF,^59^ the amount of CaCl_2_ determines a clear variation in the mechanical performance of the analyzed samples, which are overall characterized by high deformation at break and low elastic modulus. In comparison with the salt-free SS/PVA film, where the Young’s modulus and elongation at break reported in the literature are 428.47 MPa and 13.77%, respectively,^23^ the addition of calcium ions as plasticizer agent allows to match the mechanical properties of the fabricated films with those of the human skin (∼4 MPa^62^). The proposed materials exhibit superior mechanical properties compared to the most recently reported self-adhesive polymeric ECG electrodes.^63,64^

### 3.3 Ionic Conductivity

EIS measurements have been performed on three different film samples, each of them loaded with a different concentration of CaCl_2_, from 10 to 30%.

The samples were first dried in a vacuum oven at 40 °C for 1 h and then stored in an Ar-filled glovebox before the measurements. Measurements have been carried out at different, fixed relative humidity (RH) values, acquiring impedance spectra every 30 min to assess the time needed to achieve an equilibrium condition. The analysis was performed by fixing RH values from lower to higher ones and then decreasing them from higher to lower values, with the aim of examining possible hysteretic phenomena. It is worth noting that the time required to achieve equilibrium is remarkably long: excluding low RH values, it ranges from 3 to about 10 h for all samples. For clarity, due to the considerable amount of acquired data, only a representative Nyquist plot, i.e., the imaginary part of the total complex impedance *Z* (=Re*Z* + *i*(Im*Z*)) as a function of its real part, will be shown hereinafter.

**Figure 3:**
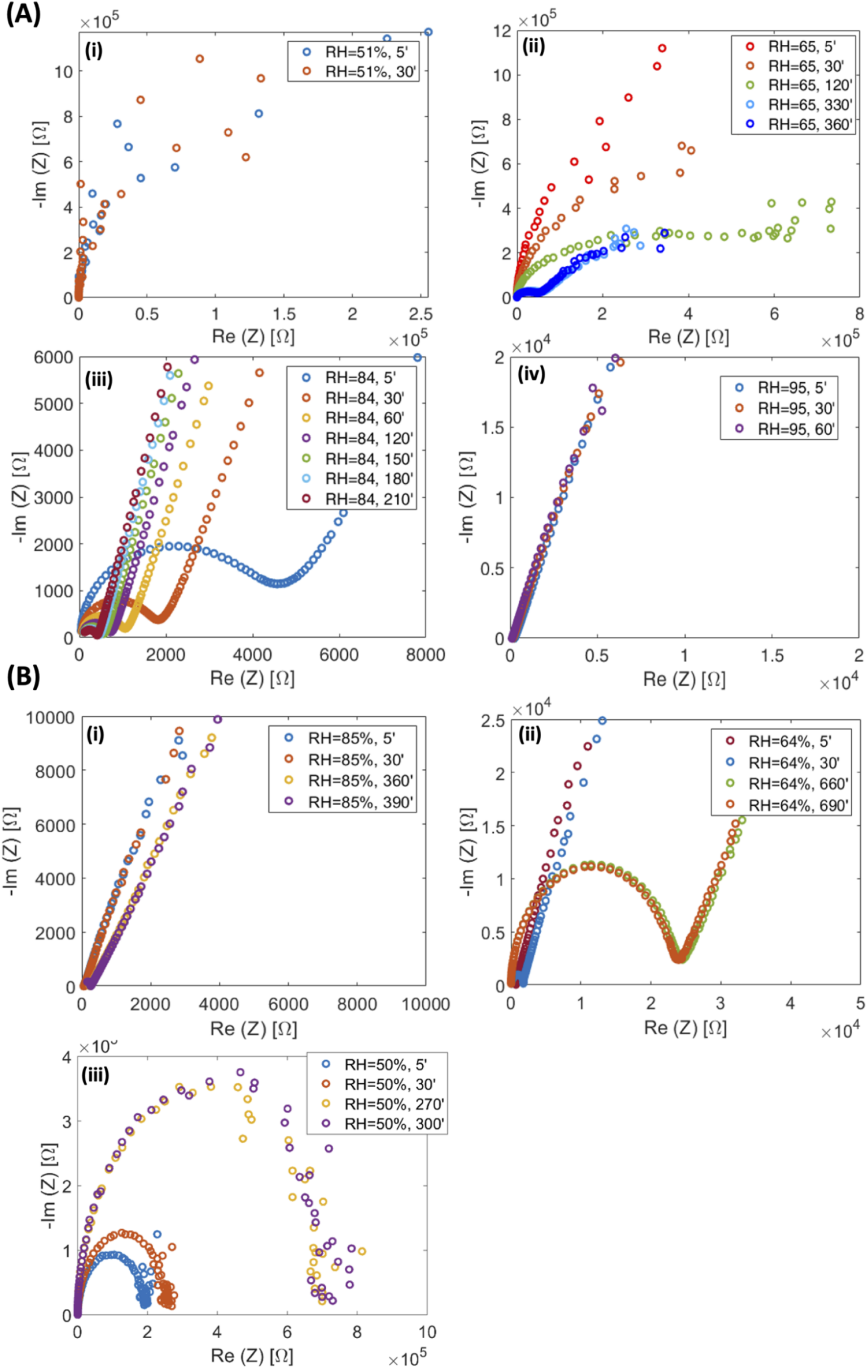
(A) SS/PVA/CaCl_2_ 20 wt % at different RH (i) 51%, (ii) 65%, (iii) 85%, and (iv) 95% while increasing humidity. (B) SS/PVA/CaCl_2_ 20 wt % at different RH (i) 85%, (ii) 64%, (iii) 50%, while decreasing humidity.

Depending on the RH, all samples are characterized by three different regimes. At low humidity conditions, CaCl_2_-loaded samples exhibited low conductivity, as demonstrated by the large semicircles in the Nyquist plots reported in Figures 3A(i–iv)–B(i–iii) and S1–S4. The absence of a linear behavior at low frequencies in the Im*Z* vs Re*Z* plot, which is a typical signature for ionic conductors, is a consequence of the low ionic conductivity of the analyzed samples. As the RH increases, semicircles in the Nyquist plot shift to higher frequencies and become smaller, indicating an increase in ionic conductivity. A linear dependence of Im*Z* as a function of Re*Z* also appears at lower frequencies, proving the ionic conduction capability of CaCl_2_-loaded SS/PVA films. Finally, in the range of higher RH, ionic resistance decreases, and the semicircles become too small to be observable; conversely, only linearity of Im*Z* as a function of Re*Z* is observed almost all along the analyzed frequency window, meaning that the ion accumulation at the interface between the probing electrodes and the SS/PVA/CaCl_2_ interface becomes prevalent, as expected in a regime of high ionic conduction. By decreasing RH, albeit a slight hysteresis, reversibility of the ionic conductivity has been observed for all samples.

In more detail, SS/PVA/CaCl_2_ samples loaded with 10 wt % CaCl_2_ are characterized by the lowest ionic conductivity (Figures S1 and S2). For RH values up to 50%, only a partial semicircle may be observed in the Nyquist plots. At RH = 65%, a whole semicircle is observed, while no linear dependence of ImZ as a function of ReZ is evidenced for frequencies of the probing AC signal varying in the window from 0.1 Hz to 100 kHz. The appearance of the low-frequency linear behavior waiting for ionic conductors is indeed observed at higher RH, starting from 84%. SS/PVA/CaCl_2_ blends loaded with a 20 wt % of CaCl_2_ contextually show a higher conductivity than that of SS/PVA/CaCl_2_ blends loaded with a lower salt concentration, as reported in Figure 3A(i–iv): here, in particular, it is shown that a lower RH is needed for 20 wt % CaCl_2_-loaded samples to achieve conductivity values comparable to those assessed for samples loaded with a 10 wt % of CaCl_2_. For these samples, evidence of ionic conductivity can be observed from RH = 65%. Moreover, at the highest RH value of 95%, the semicircles are again suppressed (ionic resistance, *R*_i_ < 1 Ω) by the dominating linear behavior in the Nyquist plot, which we remind to be ascribable to an efficient ionic conduction. As relative humidity (RH) decreases, a transition in impedance behavior is observed. Initially, at high RH, the impedance spectra exhibit a linear trend characteristic of ionic conduction. However, upon further reduction in RH, a semicircle emerges in the impedance spectra, as depicted in Figure 3B(i–iii). Finally, SS/PVA/CaCl_2_ blends with a CaCl_2_ loading of 30 wt % are characterized by the highest ionic conductivity, as evidenced by the Nyquist plots reported in Figures S3 and S4. The presence of a semicircle is already hinted for a RH percentage of 33%, although the evidence of an ionic conduction is observed for RH = 66%, while for higher RH values (i.e., from RH = 81%), due to a reduction of *R*_i_, as usual the semicircle’s size becomes small as they may not be observed anymore.

**Figure 4:**
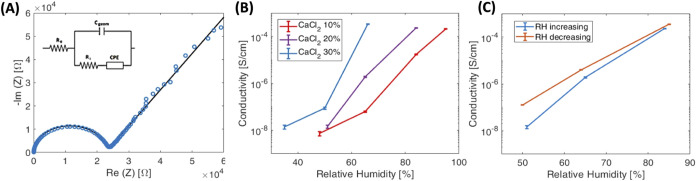
(A) Example of data fitting, performed on CaCl_2_ 20%-loaded SS/PVA at RH = 64% (inset: equivalent circuit used for modeling the ionic conduction; *R*_e_ represents the resistance of the contacts, *C*_geom_ the geometry capacitor, *R*_i_ the ionic resistance of the material under test and CPE is the constant phase element). (B) Ionic conductivity vs RH for SS/PVA/CaCl_2_ blends loaded with different weight percentage contents of CaCl_2_ (namely, 10, 20, and 30 wt %). (C) Conductivity as a function of RH.

EIS data were quantitatively analyzed by using an equivalent circuit model, as described in the Materials and Methods Section, in order to assess ionic conductivity values for the analyzed samples. An example of EIS data analysis is shown in Figure 4A, with the equivalent circuit model used to fit the acquired data reported in the inset of the figure. Ionic conductivity values, which are specifically calculated from *R*_i_ values extracted from the fitting procedure, have been reported as a function of RH in Figure 4B. The highest ionic conductivity of (3.36 ± 0.09)·10^–4^ S/cm was assessed at an RH of 66% for the sample SS/PVA/CaCl_2_ with 30 wt % of CaCl_2_, which is reasonable for the expected dependence of ionic conduction on RH. An even higher conductivity, although not quantifiable with the chosen experimental setup, is expected for higher RH. Considering both the high conductivity value at room temperature and at mild relative humidity, SS/PVA/CaCl_2_ compares favorably to other ionic conductors, in which similar conductivity values have been obtained at a higher temperature,^65^ higher humidity,^66^ or in similar systems that require soaking with a liquid electrolyte to promote efficient ionic conduction.^67,68^ A comprehensive list of conductivity values for the three types of blends under analysis at each investigated RH is available in the Supporting Information (Tables S2, S3, and S4).

The above findings all clearly evidence that the ionic conduction shown by the SS/PVA/CaCl_2_ blends is mediated by water, i.e., is assisted by the films’ water content determined by the moisture absorption. Indeed, the porous character of SS films (see SEM images reported in Figure S5) acts as a template in which water absorption occurs, allowing the mobility of Ca^2+^ and Cl^–^ ions to increase^67^ This is supported by the long time needed by the blend to reach an equilibrium condition, due to the dynamics of water absorption through inner pores; moreover, a slight hysteresis can be observed while decreasing RH, probably because some water molecules coordinate calcium cations in the blend during measurements at progressively increasing RH values (Figure 4C).

### 3.4 Skin/Electrode Impedance

**Figure 5:**
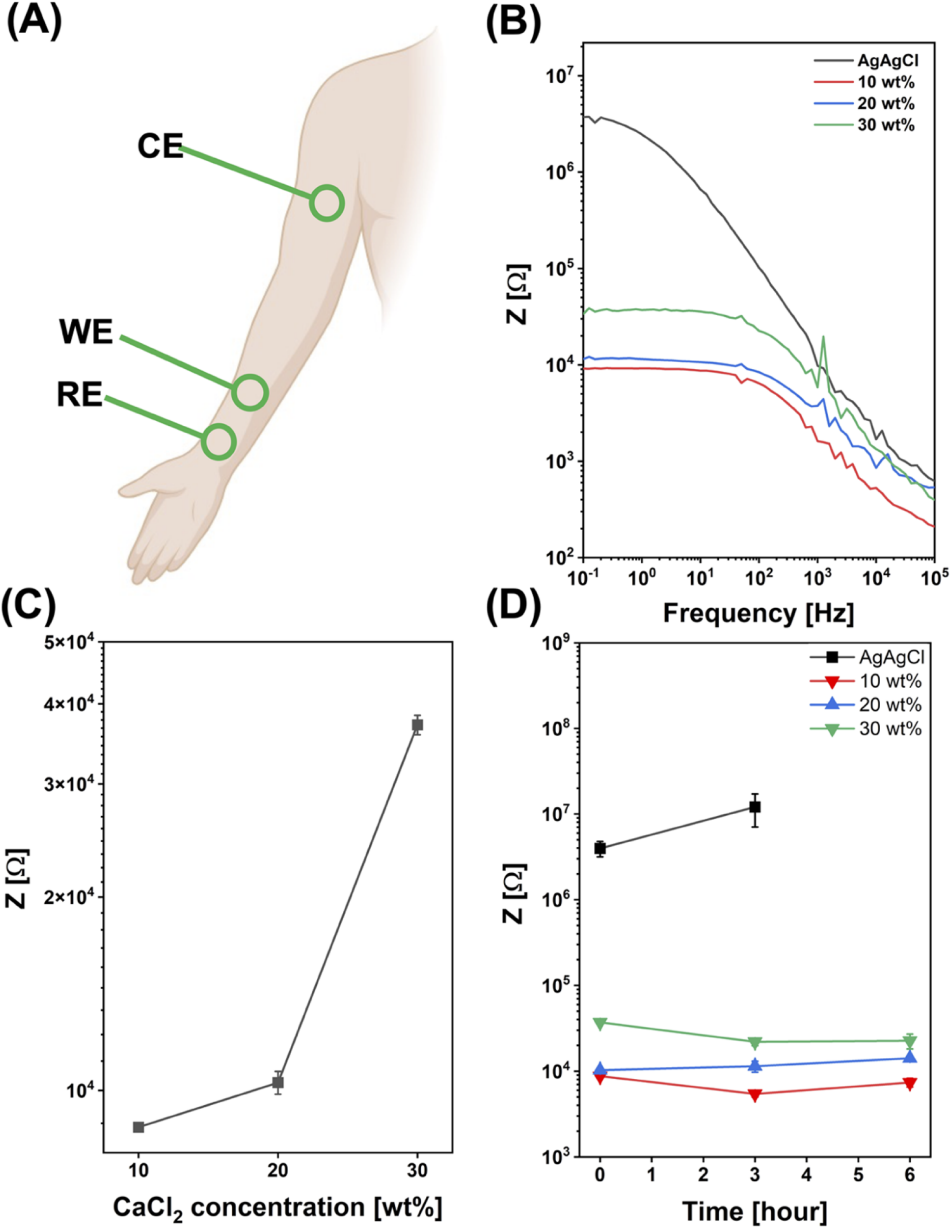
(A) Schematic of the three-electrode configuration used for skin/electrode impedance measurement. (B) Comparison among the *Z* amplitude as a function of frequency plots acquired for commercial gelled Ag/AgCl (black curve), SS/PVA/CaCl_2_ 10 wt % (red curve), SS/PVA/CaCl_2_ 20 wt % (blue curve), and SS/PVA/CaCl_2_ 30 wt % (green curve) at *t* = 0. (C) Dependence of Z at 1 Hz to the CaCl_2_ concentration (10, 20, and 30 wt %) in SS-based electrodes. (D) Time-stability of Ag/AgCl, SS/PVA/CaCl_2_ 10 wt %, SS/PVA/CaCl_2_ 20 wt %, and SS/PVA/CaCl_2_ 30 wt % over a total wearing of 6 h.

Skin/electrode impedance has been measured for the three SS composite-based electrodes under analysis and compared to that of a commercial gelled Ag/AgCl electrode. To this aim, a three-electrode configuration was used (Figure 5A), with commercial gelled Ag/AgCl electrodes serving as the counter (CE) and reference (RE) electrodes in all cases, being the CE and RE well adhered to the skin through an adhesive tape for preserving their long-lasting adhesion to the skin, while the working electrodes (WE) were again gelled Ag/AgCl electrodes and the three types of SS/PVA/CaCl_2_ films integrating Ag/AgCl snaps, respectively. Skin/electrode impedance is associated with the electrical properties of the electrode-skin interface and is a critical factor in terms of the influence it may have on the performance of epidermal electrodes.

The magnitude and stability of impedance during the electrodes lifetime are a prerogative for acquiring good quality physiological signals.^69^ Hence, reducing skin/electrode impedance values is crucial in the field of epidermal electrodes.

A comparison between the proposed electrodes and the commercial Ag/AgCl electrode at *t* = 0, i.e., just after the application of the electrodes on the skin, is shown in Figure 5B. For a better visualization, a reference is made to Figure S6A,B, where a comparison among *Z* values of the four analyzed electrodes, as measured after 3 and 6 h, is shown.

A significant reduction in impedance values (i.e., 2 orders of magnitude) between the commercial electrode and the SS-based ones is observed. This impedance reduction may be attributed to the presence of calcium ions in the SS-based films. These ions, in fact, can coordinate the moisture from the skin and external environment, increasing film adhesion and creating a better conformal contact between the skin and electrodes, thereby reducing impedance values.^70^ Additionally, water absorption is expected to induce ionic migration and to contextually increase the ionic conductivity.^59^

Figure 5C presents the *Z* value assessed at 1 Hz as a function of the CaCl_2_ concentration in the blend. The *Z* value at 1 Hz was selected for impedance comparison because it corresponds to the low limit of the frequency band filter (0.5 to 150 Hz) commonly used for ECG monitoring in clinical applications.^71^ Noteworthily, SS-based electrodes show a higher cutoff frequency of impedance, falling at the upper limit of the frequency band filter, compared to that of commercial gelled electrodes, whose cutoff frequency for impedance falls around lower frequencies of the bandpass. The behavior shown in Figure 5C reveals a slight increase of *Z* (by less than 1 order of magnitude) for an increased content of salt in the composite material. Therefore, skin/electrode impedance analysis indicates that the analyzed electrodes based on the SS blends are nearly equivalent in terms of skin/electrode impedance reduction with respect to that assessed for commercial gelled electrodes.

One of the primary challenges associated with wet electrodes is their instability over long-term monitoring, which is mainly due to the drying of the electrolytic gel. To address this issue, long-term skin/electrode impedance measurements have been carried out to evaluate the behavior of the proposed SS-PVA-CaCl_2_ electrodes in comparison with the operation features shown by commercial electrodes. Figure 5D reports a comparison of the *Z* values at 1 Hz for various electrodes as a function of the acquisition time. While the *Z* values for the SS-based electrodes remained constant after 6 h of application on the skin, the *Z* values for the Ag/AgCl gelled electrode exhibited changes in time. After 3 h of wearing, the *Z* value of the Ag/AgCl electrode increased, suggesting that the beginning of electrode detachment is underway. Additionally, the *Z* values assessed after 3 h of electrode application are less stable, as also indicated by the clearly visible increased error bar (standard deviation). Finally, the electrode detachment occurred ahead of the 6 h. In the end, the performed analysis of skin/electrode impedance suggests that (i) the SS-based electrodes are characterized by lower impedance values compared to those of commercial gelled electrodes; (ii) the concentration of calcium ions loaded in the SS films can only slightly influence impedance values, but their presence in the blend well supports the long-lasting adhesion with unchanged electrodes performance over 6 h of adhesion to the skin.

### 3.5 ECG Monitoring

**Figure 6:**
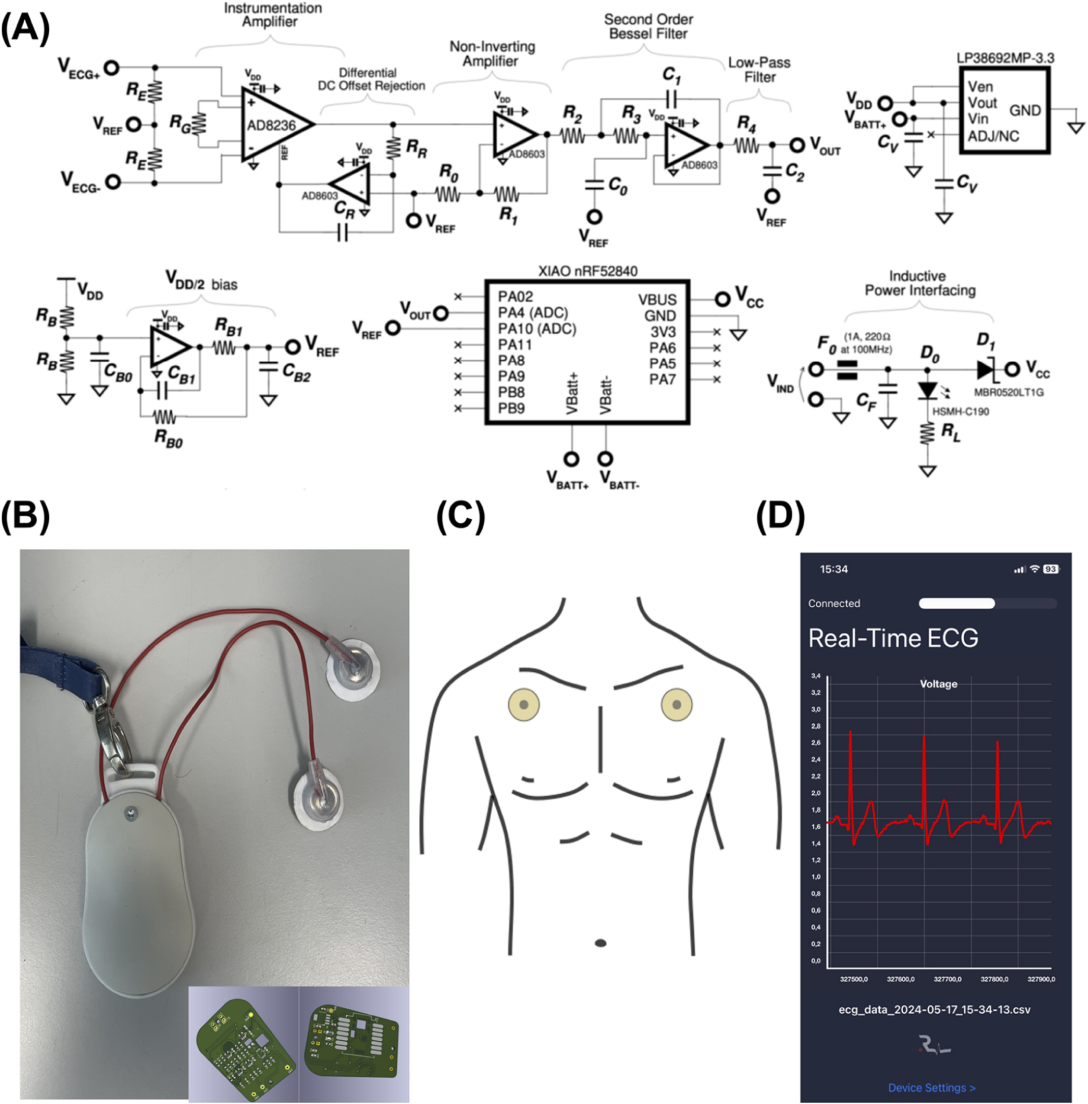
(A) Schematic of the custom Arduino-based Bluetooth ECG recorder with inductive power interfacing. (B) Photograph of the ECG Reader prototype, with customized mechanical enclosure, electrodes, and encapsulated snap connectors. In the inset, close-up of the ECG Reader PCB, top and bottom layers, with contacts to host the Seeeduino Xiao nRF52820 and holes to accommodate the battery contacts. (C) Schematic of electrode positioning on the body. (D) Screenshot of the Reader iOS application running while saving data and showing the current ECG waveform in Voltage units.

The as-prepared and characterized electrodes have finally been tested in real application. Long-lasting, continuous ECG monitoring was carried out on a healthy volunteer. A typical clinical electrocardiogram requires 12 leads, but this procedure is quite complicated to be implemented outside clinical settings and is even overkill for several applications. The eventual occurrence of dysfunctions like arrhythmia, in fact, may be identified by operating with a single lead monitoring (three electrodes).^72^ In some cases, it is possible to use only two electrodes with the third electrode in common.^73^ This procedure is usually used to reduce electrode cost and to prevent hazards from leakage currents.^74^ A custom ECG reader hardware, equipped with a modified amplifier able to work with only two electrodes, has been implemented to enable ground-free ECG monitoring. Figure 6A shows the electrical scheme of the hardware, whereas an amplifier (AD8236) was implemented with a voltage driver supply able to operate with only two electrodes. An exhaustive description of the development of ECG reader hardware and firmware may be found in the Materials and Methods Section. Using the developed hardware (final prototype in Figures 6B and 3D scheme in the inset), ECG signal recording has been carried out upon positioning two electrodes under the shoulders, as shown in the scheme of Figure 6C. The ECG signal was visualized by using a dedicated iOS app that displays voltage versus time traces (Figure 6D). The ECG signals from SS/PVA/CaCl_2_ 10 wt %, SS/PVA/CaCl_2_ 20 wt %, and SS/PVA/CaCl_2_ 30 wt % electrodes have been continuously recorded for 1 h and compared to those from commercial gelled Ag/AgCl electrodes. An example of ECG signals acquired with SS electrodes is shown in Figure 6A, while the comparison of the ECG signals is shown in Figure S7A–C, whereas 6 s-long windows reporting signals at the start of the acquisition (*t* = 0), after 30 min and after 1 h, are reported, respectively.

**Figure 7:**
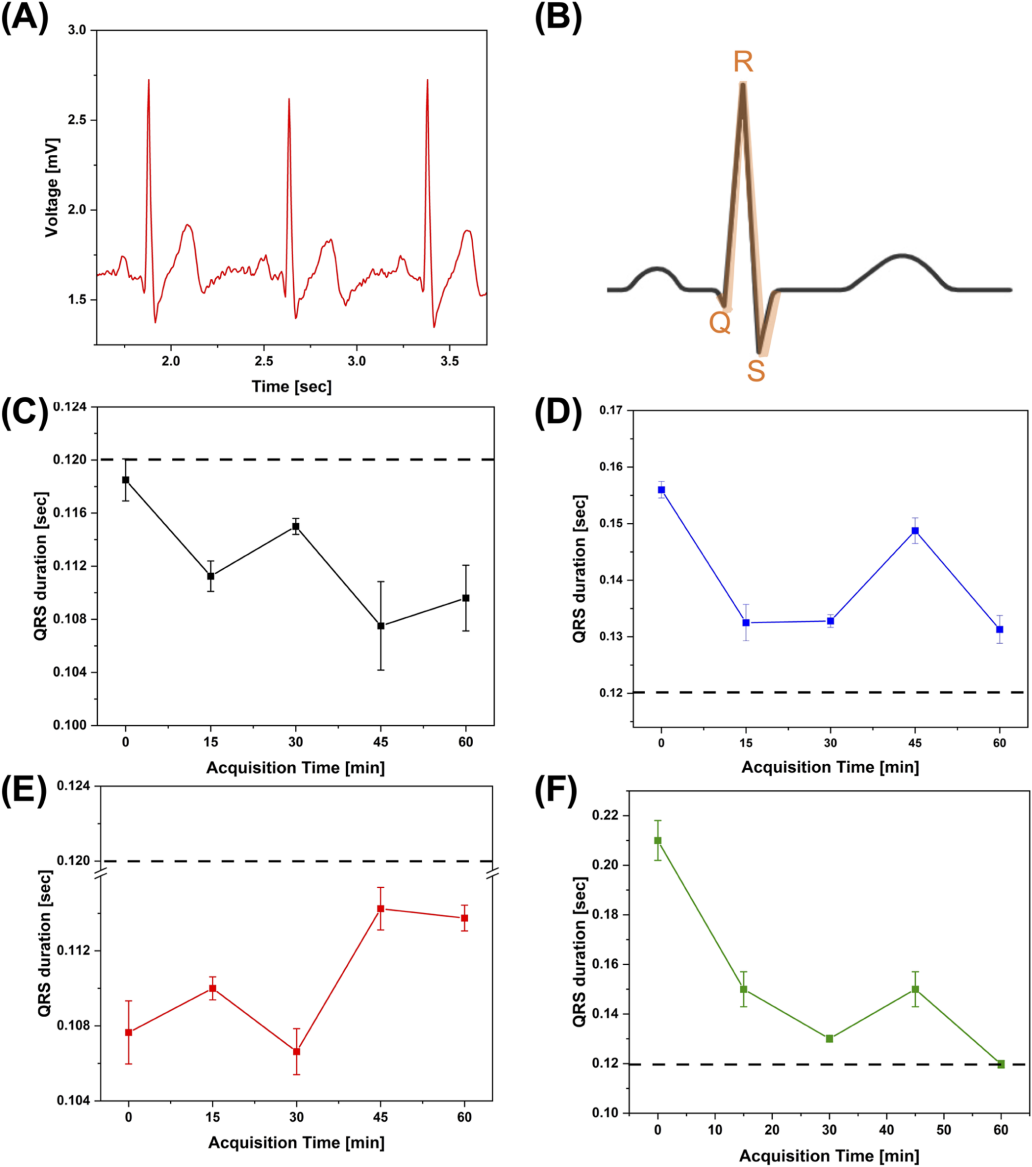
(A) Typical ECG waveforms acquired using a SS/PVACaCl_2_ electrode; (B) schematic of a typical QRS peak; QRS duration as a function of time monitoring of (C) commercial Ag/AgCl QRS, (D) SS/PVA/CaCl_2_ 10 wt %, (E) SS/PVA/CaCl_2_ 20 wt %, and (F) SS/PVA/CaCl_2_ 30 wt % (dotted lines indicate the threshold clinical value of QRS duration for healthy people, i.e., 0.12 s).

An ECG signal is characterized by three main features: the P wave, associated with atrial depolarization; the QRS complex, reflecting ventricular depolarization; and the T wave, representing ventricular depolarization. QRS peak is the most intense one in ECG waveforms (Figure 7B), and an analysis of its duration is statistically associated to heart failure, whereas a short duration indicates a rapid depolarization of ventricles while wider QRS peaks may be indicative of dysfunctions in the conduction system of the heart.

The characteristics of the QRS peak have been analyzed to figure out the composition of SS electrodes realizing an ECG waveform comparable to that of commercial gelled electrodes.^75,76^Figure 7C–F shows the QRS duration as a function of monitoring time for each electrode under testing. The dotted lines indicate the clinical value of QRS time width for healthy people, which does not exceed 0.12 s.^77^

Electrodes displaying values of the QRS peak width that are above 0.12 s may not be considered usable in clinical applications. In this respect, the QRS duration calculated from ECG recorded with commercial gelled electrodes (Figure 7C) is below this value, and small fluctuations upon continuous monitoring over long time scales are expected for commercial devices. Nevertheless, variations of a few milliseconds are not relevant and are completely negligible for clinical purposes. The same result has been found for the SS/PVA/CaCl_2_ 20 wt % (Figure 7E), while SS/PVA/CaCl_2_ 10 wt % and SS/PVA/CaCl_2_ 30 wt % are characterized by a QRS duration above the clinical limit (Figure 7D–F).

We infer that an amount of salt concentration of 10 wt % in the SS/PVA/CaCl_2_ blend is not enough to allow an efficient absorption of moisture, and the electrodes are contextually characterized by a weaker adhesion due to fast drying during the continuous measurements. Conversely, for a salt content of 30 wt %, the moisture absorption, in all likelihood, is excessive, and the excess of water may also cause the electrode to slip over the skin due to swelling effects occurring during the running measurements. Moreover, it is known that drying effects in time may affect the total electrode impedance causing a faster increase of its value in case of higher ionic content around the Ag/AgCl active area.^78^

Considering the healthy state of the volunteer, only the SS/PVA electrodes loaded with 20 wt % of CaCl_2_ could be considered acceptable in clinical applications, showing a QRS duration falling in the range of normal values. SS/PVA/CaCl_2_ with 20 wt % of salt content were tested in long-term ECG monitoring, i.e., up to 6 h, demonstrating the ability of these electrodes for prolonged acquisition, as shown in Figure S8. Overall, it is important to consider that these results depend on several factors such as environmental humidity, amount of sweat, and body temperature of the subjects. The good quality of ECG signals preserved upon long-lasting adhesion to the skin indeed is at least indicative of negligible detrimental effects on the overall electrode performance induced by a possible reduction of adhesion strength or even by its partial detachment.

## 4 Conclusions

This work has demonstrated a strategy toward the assembly of gel-free electrodes with self-adhesive properties suited for ECG trace recording and unobtrusive, continuous monitoring of biopotentials with a stable performance in time. The proposed electrodes are based on a biofilm of animal origin (SS) that is modified by a biocompatible additive (PVA) and a hygroscopic salt (CaCl_2_), all components mixed in different concentration ratios. An extensive characterization has been performed on SS/PVA blends (1:1 v/v mixing, accounting for a proper film forming) with 10, 20, and 30 wt % of CaCl_2_. The chemical characterization has shown that calcium ions in well-mixed blends (i.e., no phase separation is evidenced upon morphological analysis by SEM) efficiently coordinate water molecules from environmental and skin humidity, thus promoting adhesive features in related films because of the high calcium coordination number (from 6 to 8 water molecules in the first solvation shell). The mechanical analysis has indicated that Young’s moduli extracted for all of the analyzed blends, falling in the range of units/tens of MPa, are comparable to that of skin, while a far enhanced elongation at break (from 250 to 450%) with respect to that of CaCl_2_-free SS/PVA blends (around 10%) may be found as well, all of these aspects determining a stable adhesion in time of blend-based films to the skin. Measurements of ionic conduction of blends as a function of relative humidity, evaluated via electrochemical analysis, have indeed shown that the blends well conduct ionic species (with a best ionic conductivity of (3.36 ± 0.09) × 10^–4^ S/cm), above all at higher environmental humidity that determines massive adsorption of water molecules and, contextually, imparts a solid electrolyte-like behavior to the blend. Moreover, direct skin/electrode impedance measurements on blend-based electrodes show a modulus of skin/electrode impedance falling in the range of 10^4^ Ω, i.e., 2 orders of magnitude lower than that of commercial ECG electrodes. These findings both corroborate the expected enhanced efficiency toward long-lasting ECG measurement sessions by the proposed blend-based electrodes. Here, the function of sustained ionic conductivity is that of electrolytic gels in commercial electrodes, while the reduced skin/electrode impedance allows a better impedance matching between the skin and the electrode.

In conclusion, the main characteristics of electrodes based on adhesive films of the blend are (i) biocompatibility and body tolerability ensured by the nature of the adhesion film’s constituents; (ii) sustainability due to the use of SS protein, a byproduct of silk production; (iii) “green” character arisen from methods (simple blending), solvents (water in this case), and the absence of cross-linking agents; and (iv) long-lasting ECG recording at least up to 6 h, as tested in this experiment, with stable impedance amplitude and, accordingly, high signal quality, as also confirmed by the analysis of QRS peak duration, which is below 0,12 s for the as-selected best-performing film formulation in terms of prolonged adhesion and impedance stability, i.e., the SS(4 wt %)/PVA(4 wt %)/CaCl_2_(20 wt %) blend.

## Supporting Information Available

SS/PVA/CaCl_2_ blend composition; EIS of SS/PVA/CaCl_2_ 10 wt % at different RH; EIS of SS/PVA/CaCl_2_ 10 wt % at different RH and conductivity; EIS of SS/PVA/CaCl_2_ 30 wt % at different RH; EIS of SS/PVA/CaCl_2_ 10 wt % at different RH and conductivity; SS/PVA/CaCl_2_ 30 wt % at different RH; SEM image of SS/PVA/CaCl_2_ 10 wt % cross section; SS/PVA/CaCl_2_ 10 wt % conductivity; SS/PVA/CaCl_2_ 20 wt % conductivity; SS/PVA/CaCl_2_ 30 wt % conductivity; EIS comparison between commercial ECG electrodes and SS/PVA/CaCl_2_ electrodes; ECG comparison between commercial ECG electrodes and SS/PVA/CaCl_2_ electrodes after 60 min; ECG acquired for 6 h (PDF)
