# Supplementary material for: Sericin Electrodes with Self-Adhesive Properties for Biosignaling
Source: ACS Biomater Sci Eng. 2025 Feb 4;11(3):1776–91. doi: 10.1021/acsbiomaterials.4c02234 (PMC12079639; doi:10.1021/acsbiomaterials.4c02234)
Supplement: Supplementary file 1 — ab4c02234_si_001.pdf [file ab4c02234_si_001.pdf]

Supplementary Information

Sericin Electrodes with Self-adhesive Properties for Bio-signaling

Davide Vurro [†], Aris Liboà [†,‡], Ilenia D'Onofrio [†,‡], Giuseppe De Giorgio [†], Silvio Scaravonati [§], Marco Crepaldi [‖], Alessandro Barcellona [‖], Corrado Sciancalepore [⊥], Vardan Galstyan [†], Daniel Milanese [⊥], Mauro Riccò[§], Pasquale D'Angelo [,†], Giuseppe Tarabella [†]*

[†] Institute of Materials for Electronics and Magnetism (IMEM-CNR) – Parco Area delle Scienze 37/A, 43124 Parma, Italy.

[‡] Dep. of Chemistry Life Sciences and Environmental Sustainability, University of Parma, Parco Area delle Scienze 17/A, 43124 Parma, Italy

[§] Dep. of Mathematical, Physical and Computer Sciences, University of Parma, GISEL & INSTM, Parco Area delle Scienze 7/A, 43124 Parma, Italy

[‖] Electronic Design Laboratory, Fondazione Istituto Italiano di Tecnologia, Via Enrico Melen 83, 16152 Genova, Italy.

[⊥] Department of Engineering for Industrial Systems and Technologies, University of Parma, Parco Area delle Scienze 181/A, 43124 Parma, Italy

* corresponding author: pasquale.dangelo@cnr.it

Table S1: SS/PVA/$CaCl_2$ blend composition (selected concentration were highlighted in green)

SS (wt%)	PVA (wt%)	$CaCl_2$ (wt%)
2	4	10
2	4	20
2	4	30
2	4	40
4	4	/
4	4	10
4	4	20
4	4	30
4	4	40

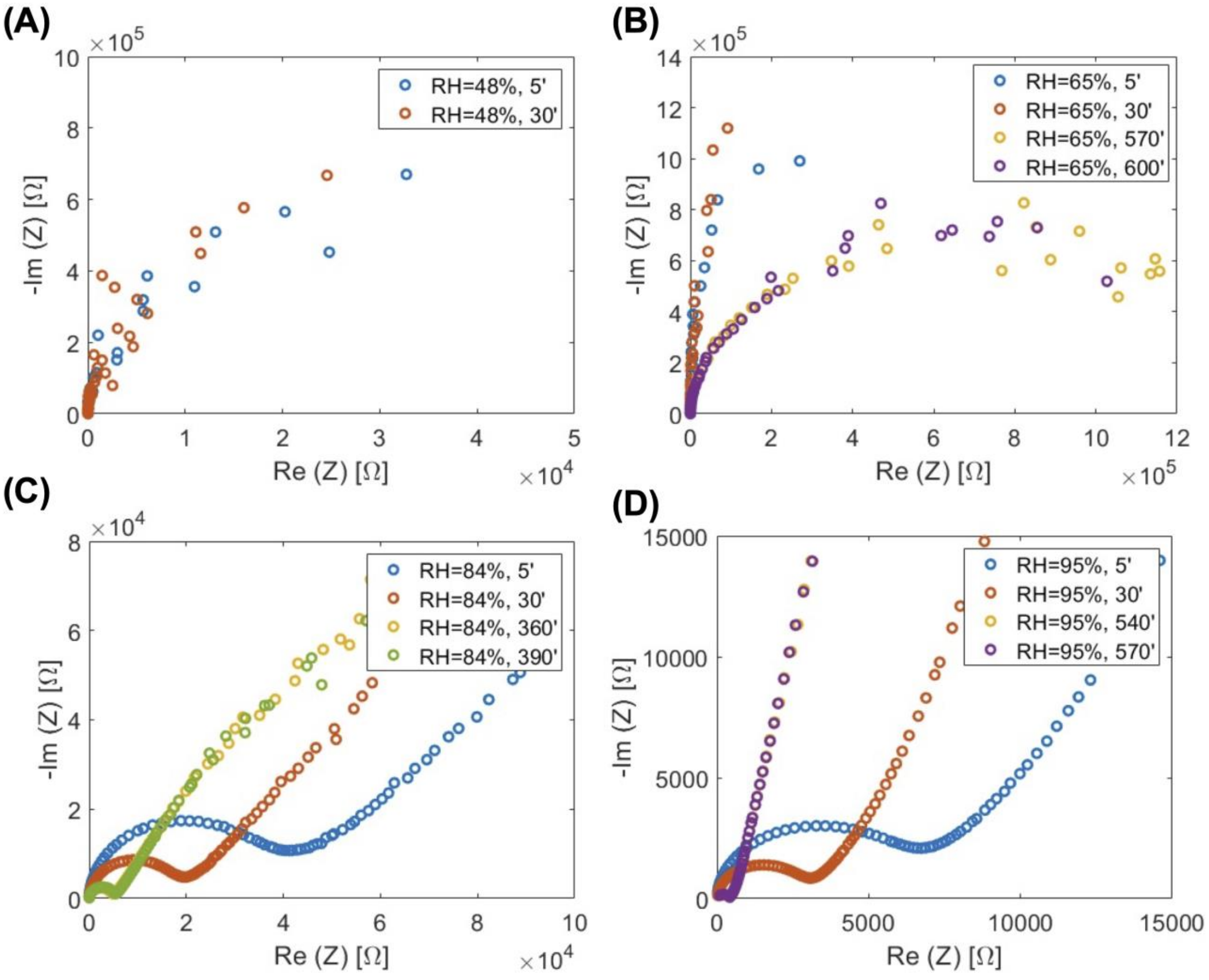

Figure S1: A, B, C, D) SS/PVA/$CaCl_2$ 10 wt% at different RH, while increasing humidity

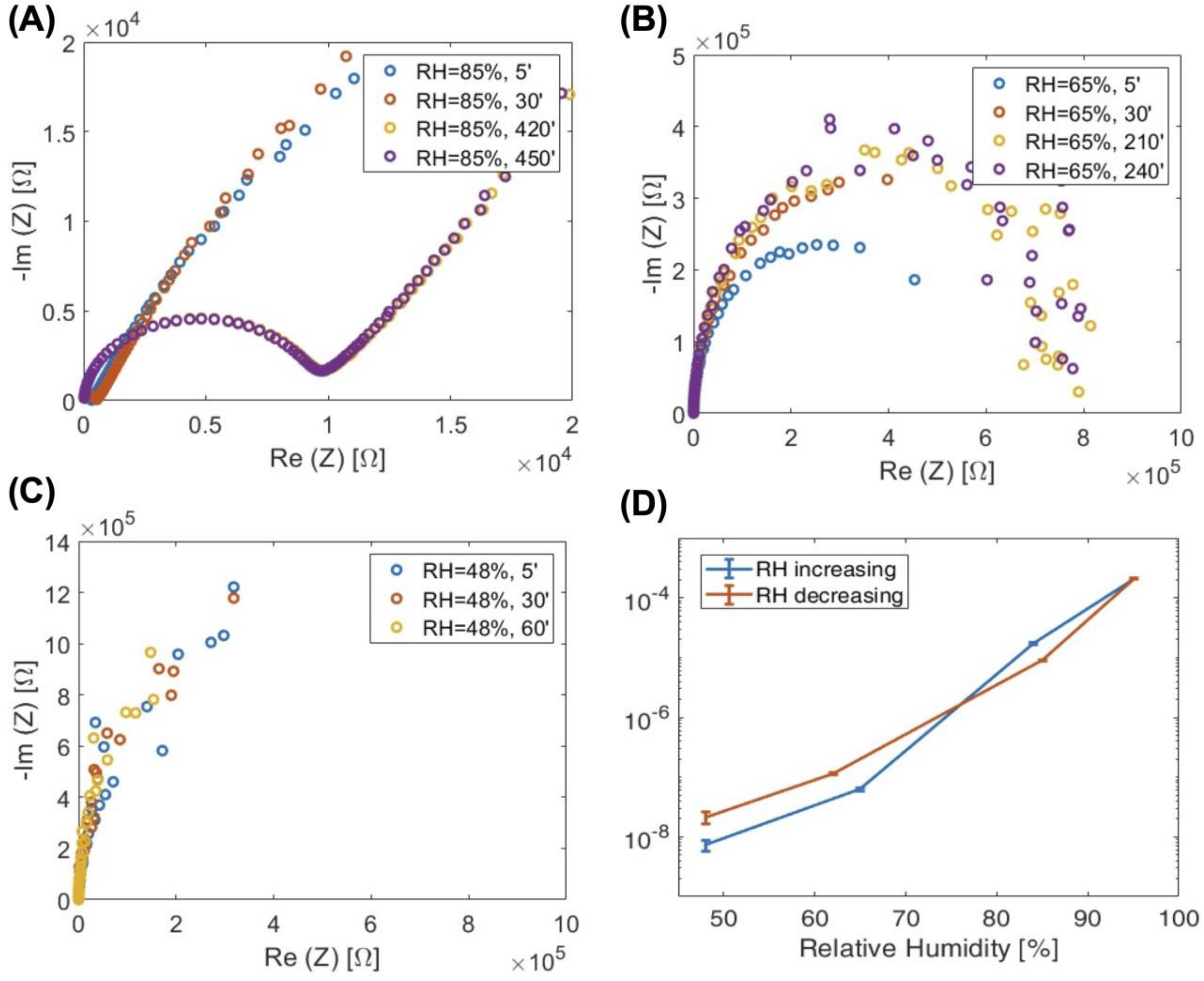

Figure S2: A, B, C) SS/PVA/$CaCl_2$ 10 wt% at different RH, while decreasing humidity, and D) conductivity as a function of RH

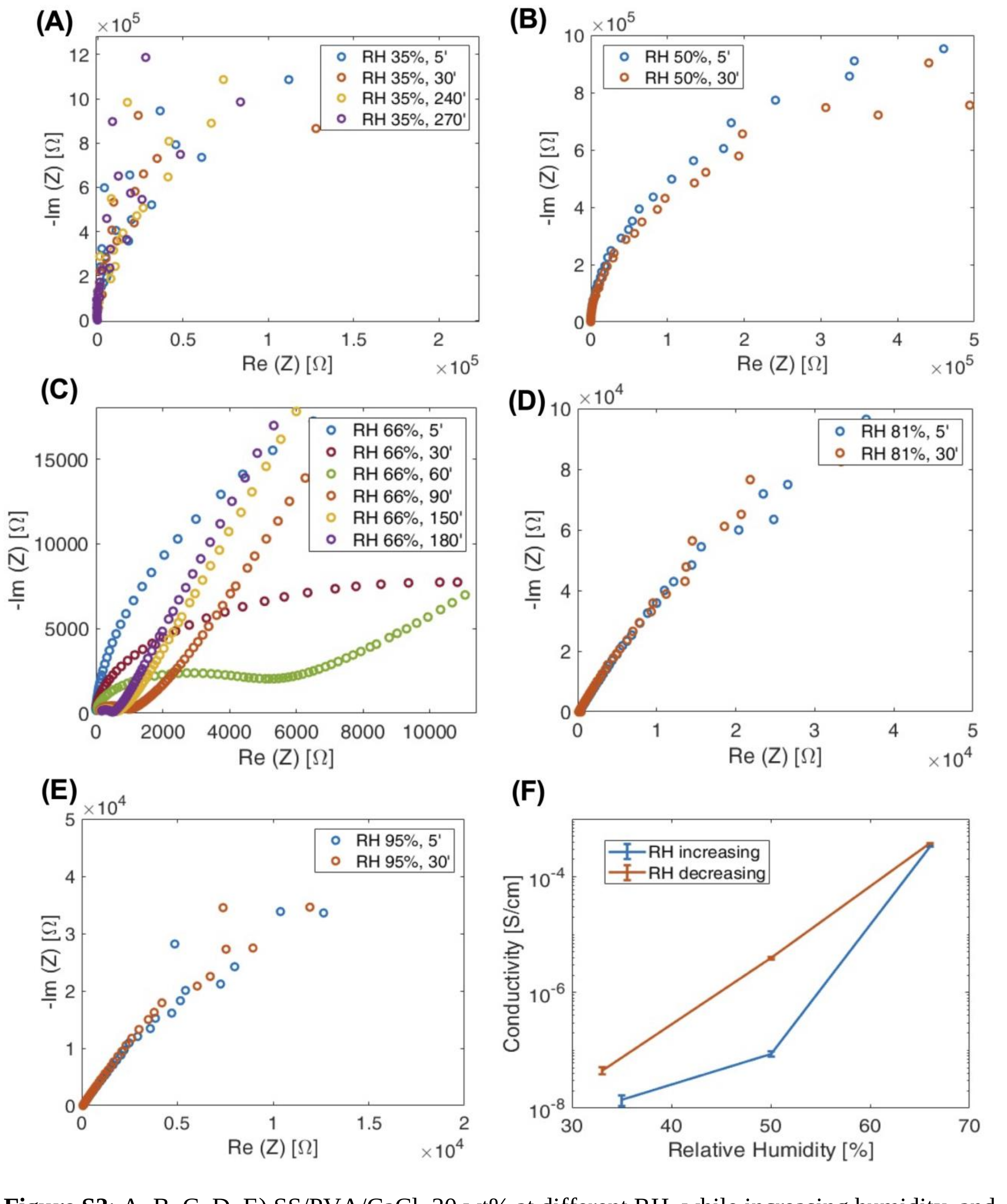

Figure S3: A, B, C, D, E) SS/PVA/$CaCl_2$ 30 wt% at different RH, while increasing humidity, and F) conductivity as a function of RH

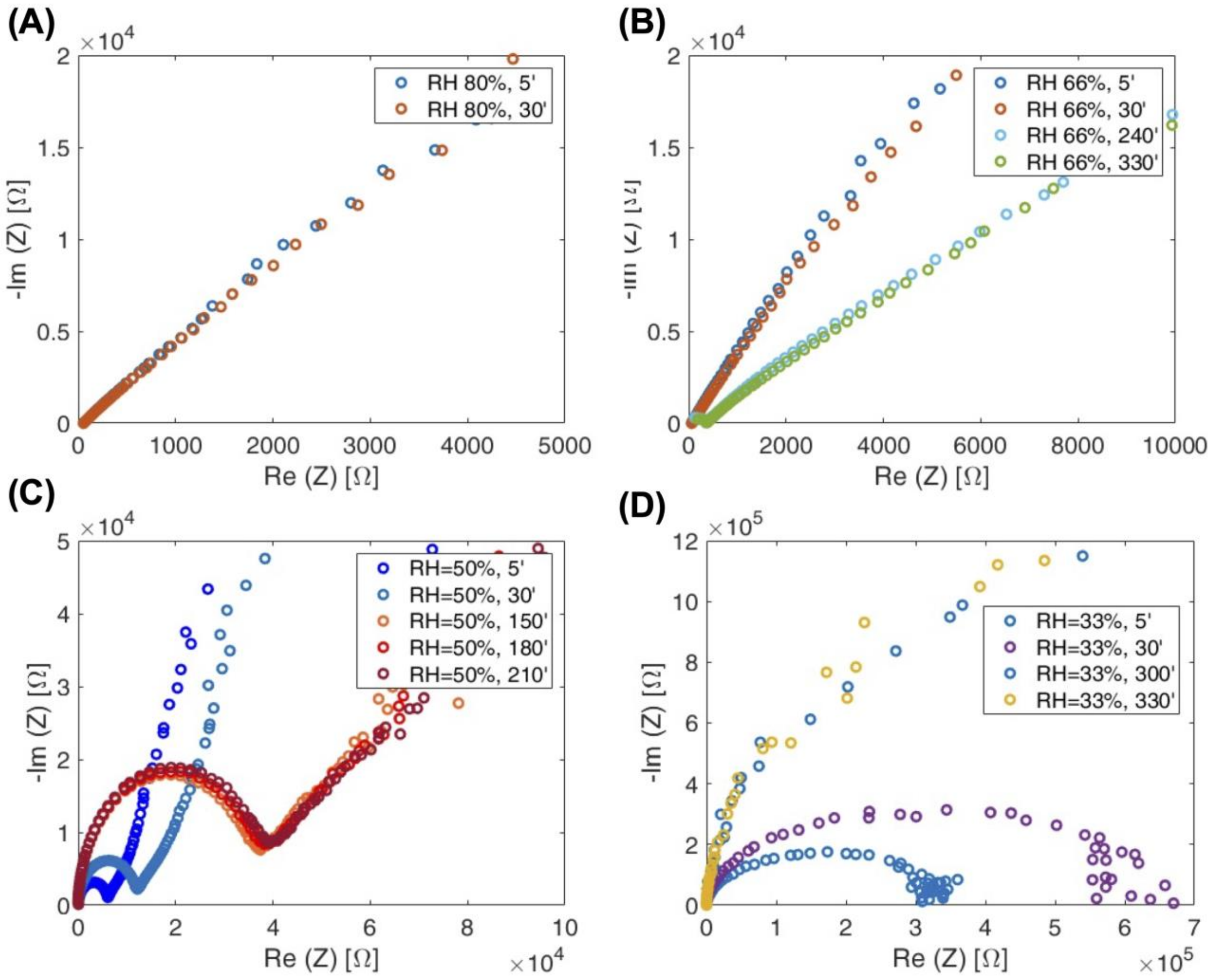

Figure S4: A, B, C, D) SS/PVA/$CaCl_2$ 30 wt% at different RH, while decreasing humidity

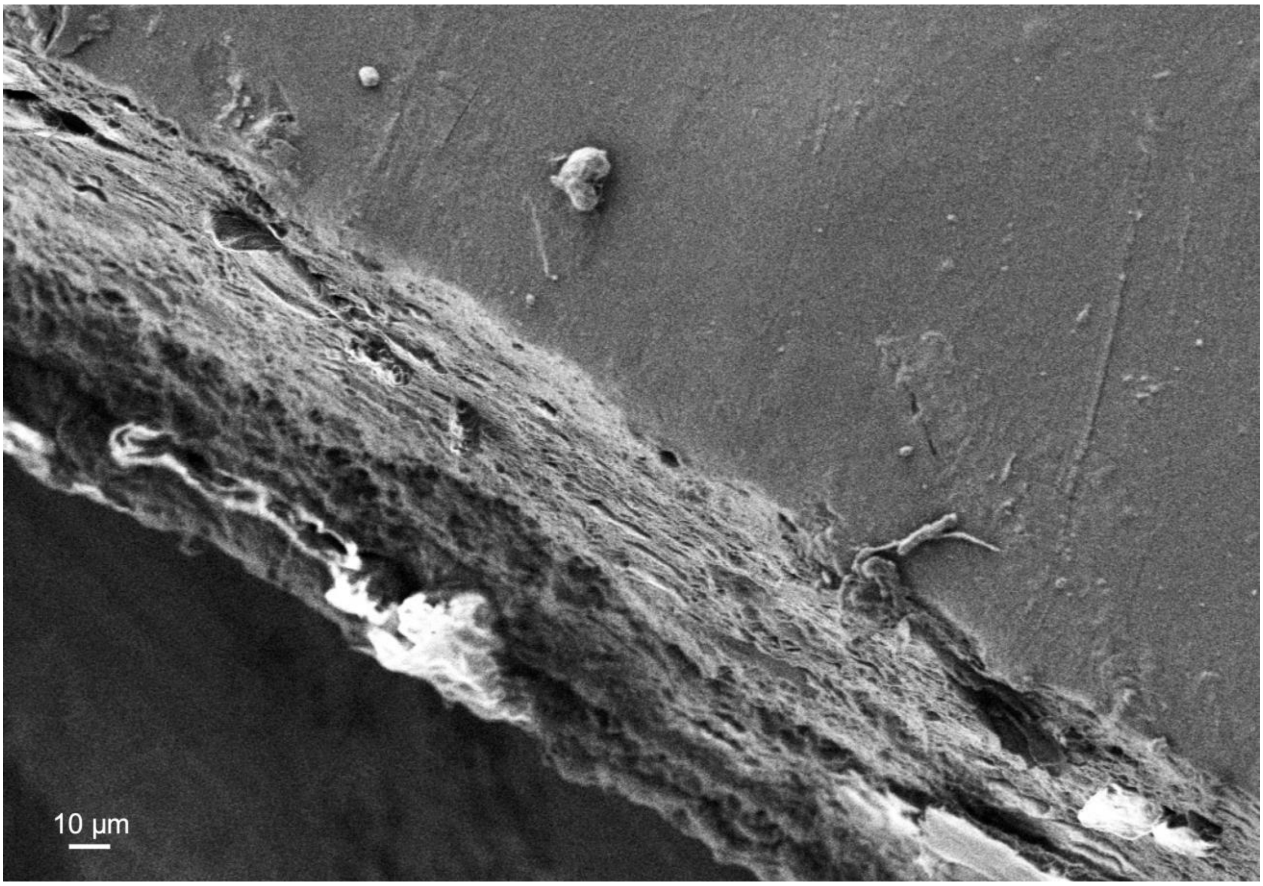

Figure S5: SEM image of SS/PVA/$CaCl_2$ 10 wt% cross section (EHT: 5KV, Mag.: 898 X, WD:7.5 mm)

Table S2: SS/PVA/$CaCl_2$ 10 wt% conductivity

RH (%)	Conductivity (S/cm)
48, increasing	$(7\pm1)\cdot10^{-9}$
65, increasing	$(6.2\pm0.4)\cdot10^{-8}$
80, increasing	$(1.70\pm0.08)\cdot10^{-5}$
95, increasing	$(2.09\pm0.06)\cdot10^{-4}$
85, decreasing	$(9.0\pm0.2)\cdot10^{-6}$
62, decreasing	$(1.14\pm0.05)\cdot10^{-7}$
48, decreasing	$(2.2\pm0.6)\cdot10^{-8}$

Table S3: SS/PVA/$CaCl_2$ 20 wt% conductivity

RH (%)	Conductivity (S/cm)
51, increasing	$(1.4\pm0.2)\cdot10^{-8}$
65, increasing	$(1.9\pm0.1)\cdot10^{-6}$
84, increasing	$(2.30\pm0.07)\cdot10^{-4}$
85, decreasing	$(3.50\pm0.05)\cdot10^{-4}$
64, decreasing	$(3.99\pm0.07)\cdot10^{-6}$
50, decreasing	$(1.26\pm0.04)\cdot10^{-7}$

Table S4: SS/PVA/$CaCl_2$ 30 wt% conductivity

RH (%)	Conductivity (S/cm)
35, increasing	(1.4±0.3) $\cdot 10^{-8}$
50, increasing	(8.7±0.9) $\cdot 10^{-8}$
66, increasing	(3.36±0.09) $\cdot 10^{-4}$
66, decreasing	(3.74±0.0.8) $\cdot 10^{-4}$
50, decreasing	(3.9±0.1) $\cdot 10^{-6}$
33, decreasing	(4.5±0.6) $\cdot 10^{-8}$

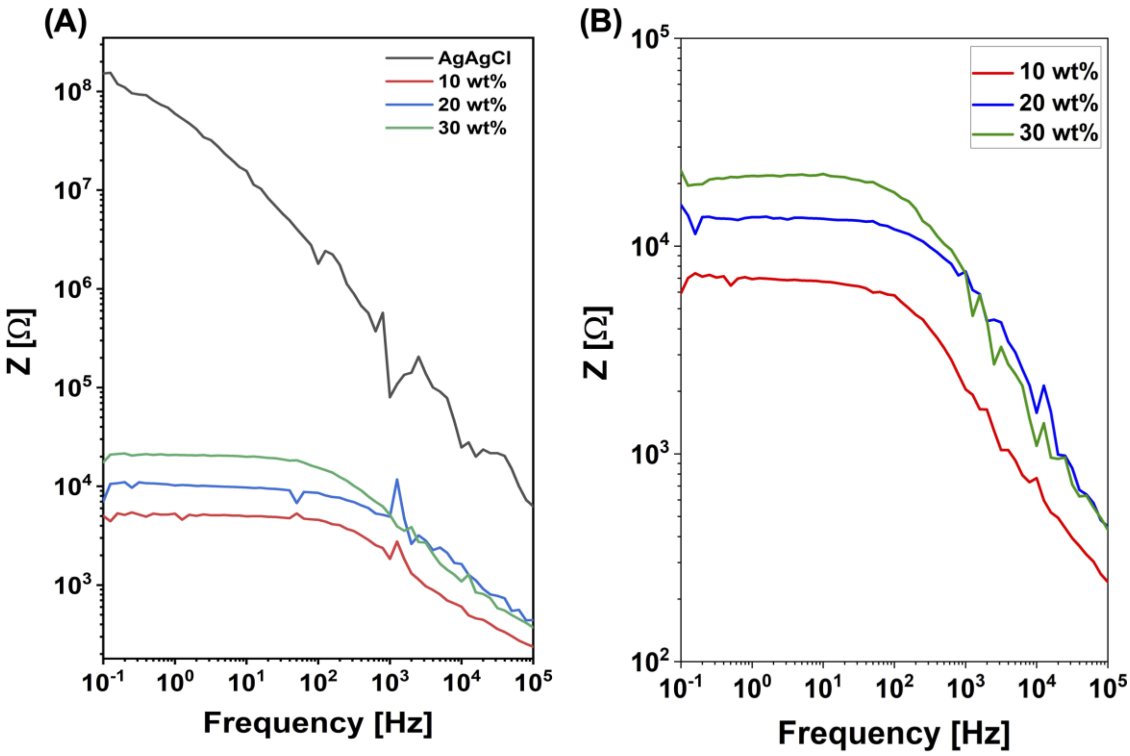

Figure S6: Comparison of the Bode plot of commercial gelled Ag/AgCl (black curve), SS/PVA/$CaCl_2$ 10 wt% (red curve), SS/PVA/$CaCl_2$ 20 wt% (blue curve) and SS/PVA/$CaCl_2$ 30 wt% (green curve) at A) t=1h and B) t= 6 h

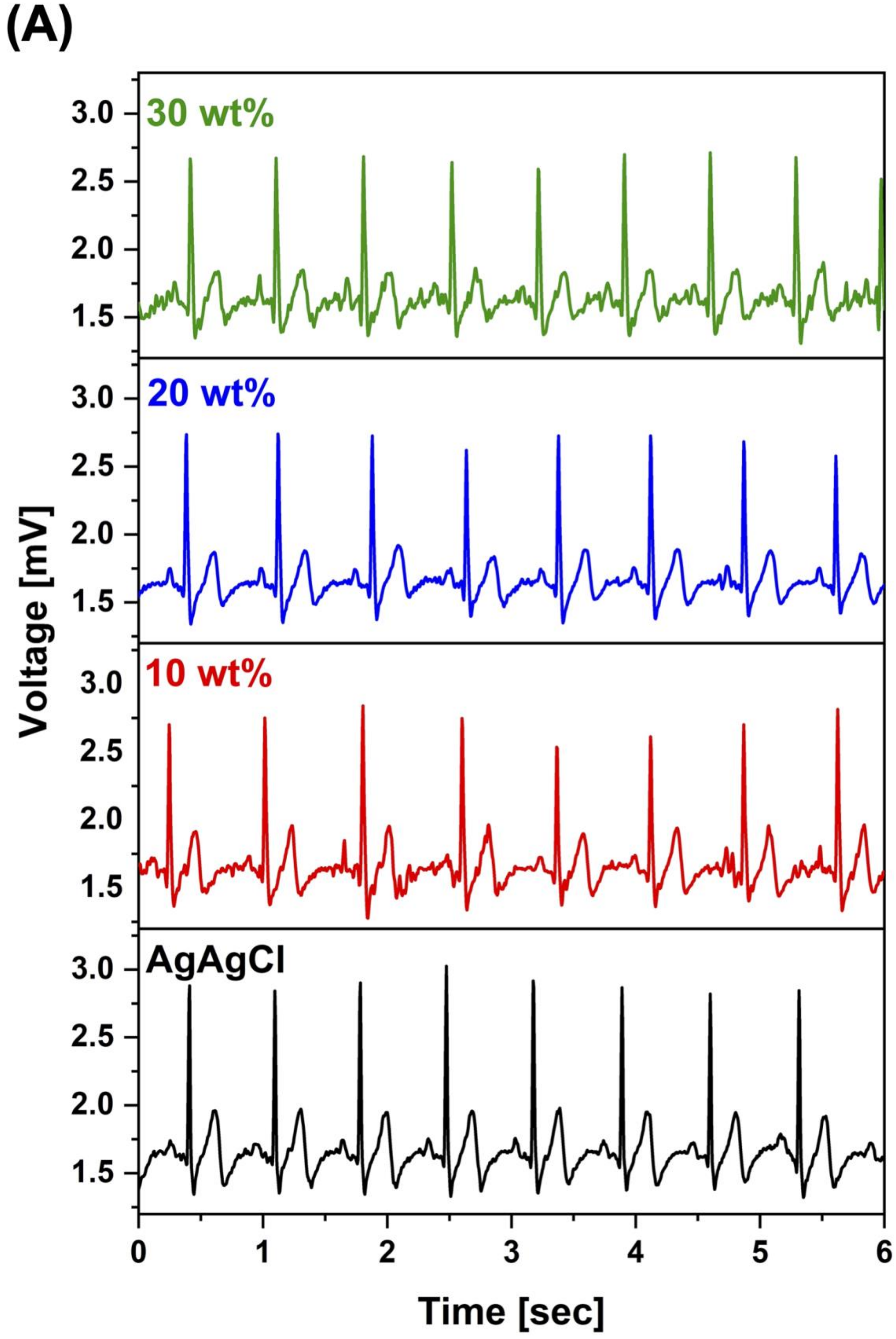

(A)
30 wt%
20 wt%
10 wt%
AgAgCl
Voltage [mV]
3.0
2.5
2.0
1.5
0
1
2
3
4
5
6
Time [sec]

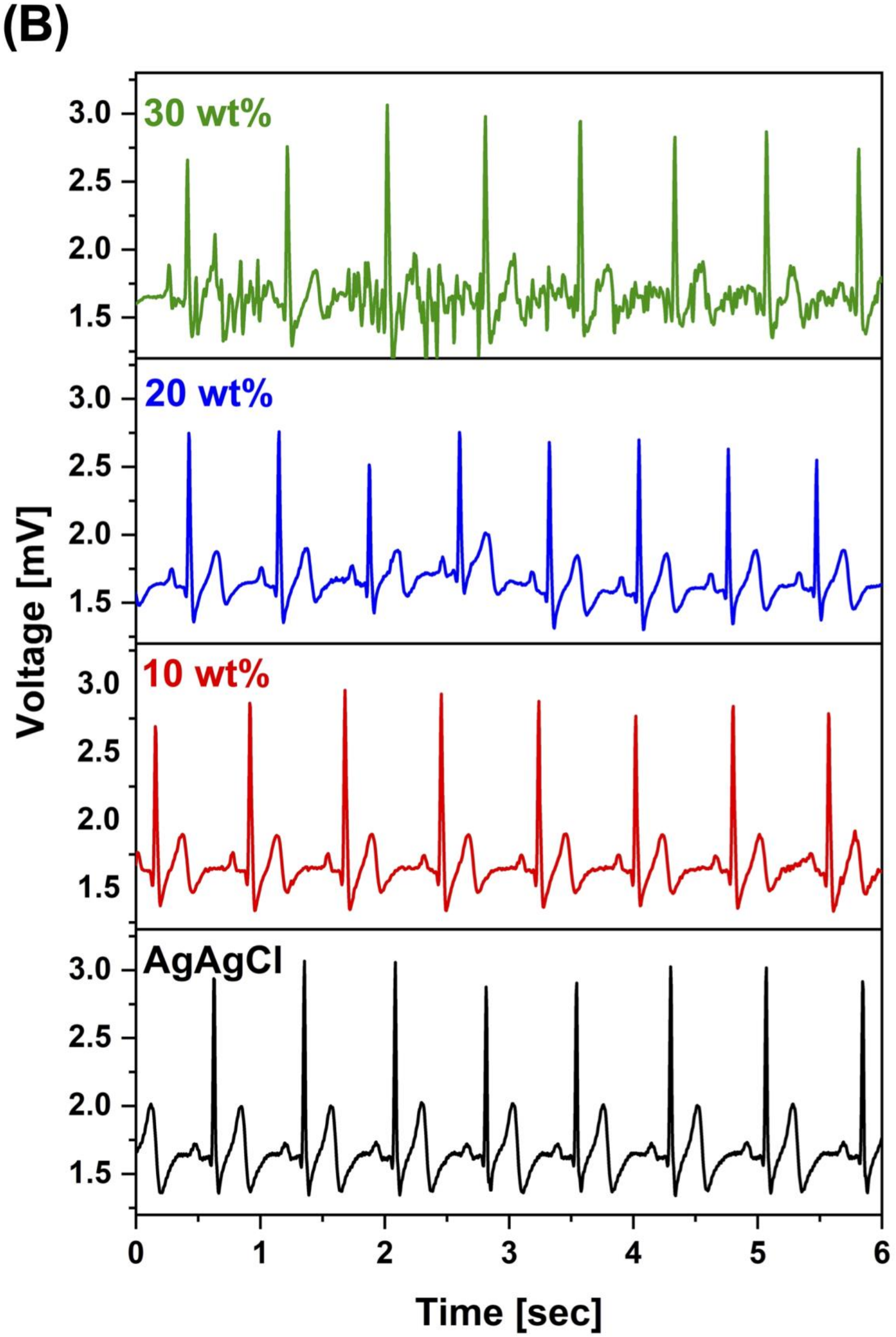
(B)
30 wt%
20 wt%
10 wt%
AgAgCl
Voltage [mV]
Time [sec]
3.0
2.5
2.0
1.5
0
1
2
3
4
5
6

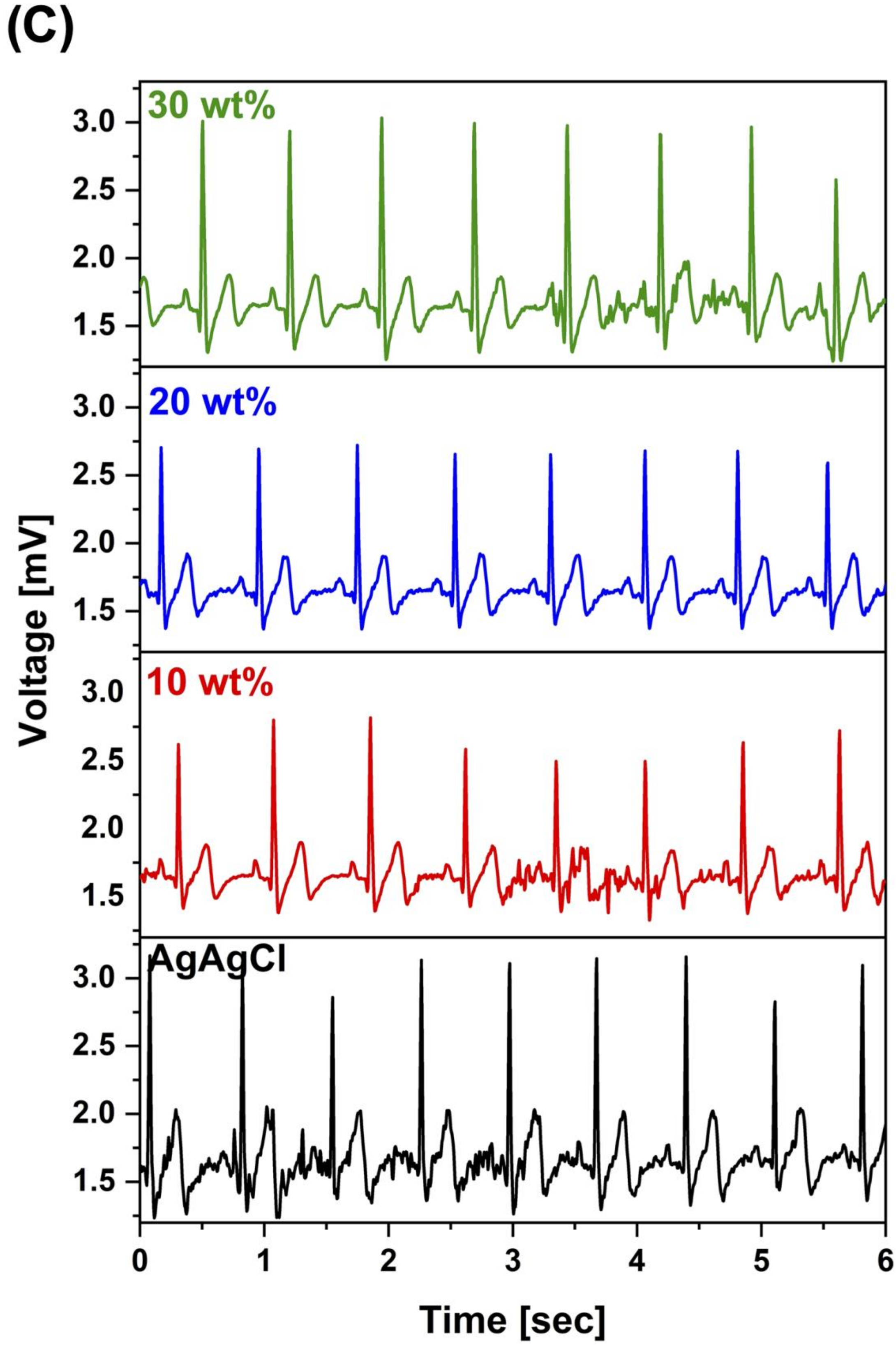

Figure S7: Comparison between ECGs signal acquired using commercial AgAgCl, SS/PVA/$CaCl_2$ 10 wt%, SS/PVA/$CaCl_2$ 20 wt% and SS/PVA/$CaCl_2$ 30 wt% at A) t =0, B) t= 30 min and C) t= 60 min

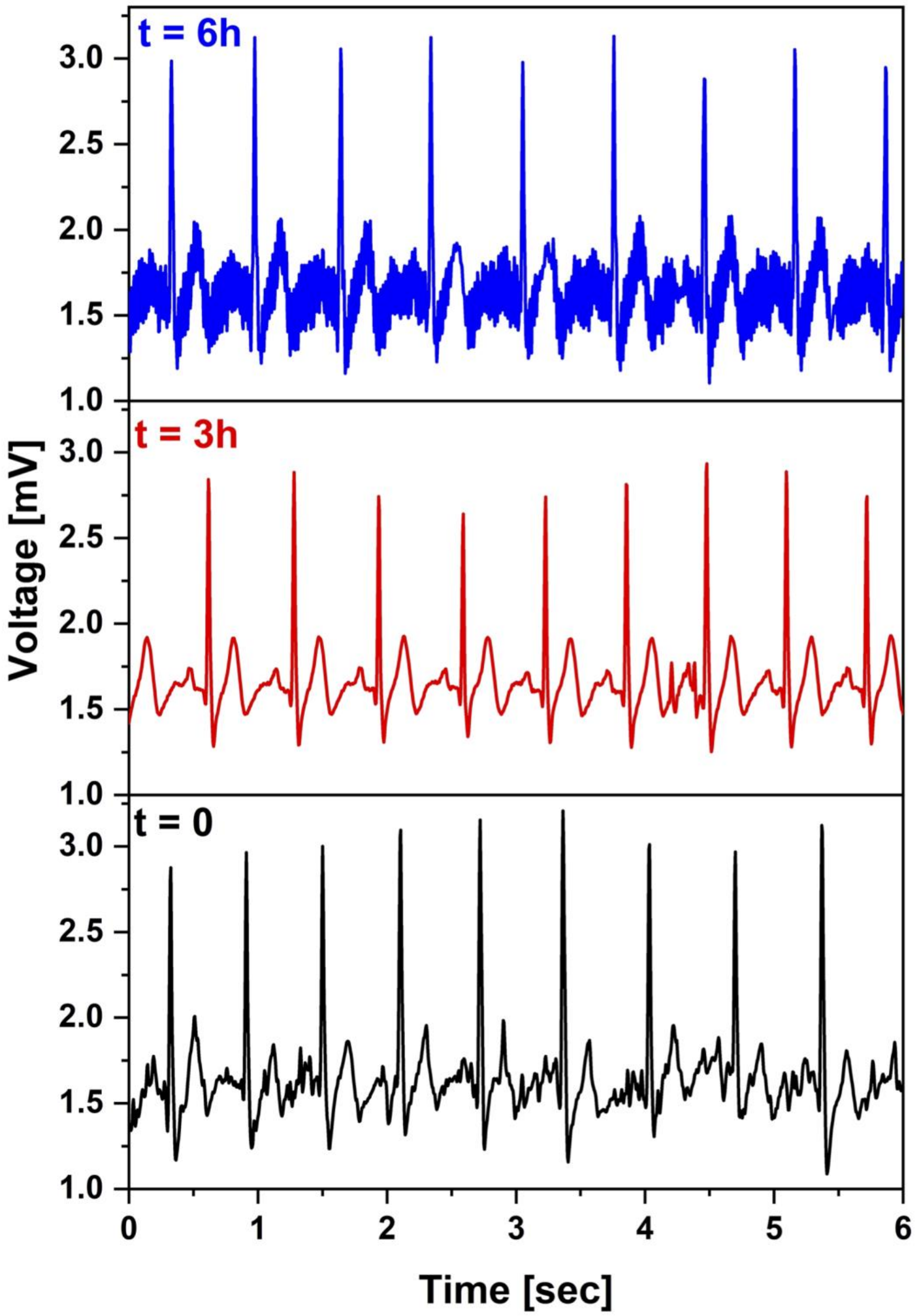

Figure S8: ECG signal acquired using SS/PVA/$CaCl_2$ 20 wt% in a long-term monitoring (6h). For a better visualization only the first 6 sec of acquisition at 0 (black), 3h (red) and 6h (blue) were reported
